# Digital Detection of Dementia in Primary Care: A Randomized Clinical Trial

**DOI:** 10.1001/jamanetworkopen.2025.42222

**Published:** 2025-11-10

**Authors:** Malaz A. Boustani, Zina Ben Miled, Arthur H. Owora, Nicole R. Fowler, Paul Dexter, Eric Puster, Randall W. Grout, Diana Summanwar, Saura Fortin Erazo, Shanell Disla, Katrina Coppedge, James E. Galvin

**Affiliations:** 1 Department of Medicine, Indiana University School of Medicine, Indianapolis; 2 Indiana University Center for Aging Research, Indianapolis; 3 Regenstrief Institute Inc, Indianapolis, Indiana; 4 Center for Health Innovation and Implementation Science, Indiana University School of Medicine, Indianapolis; 5 Eskenazi Health, Health and Hospital Cooperation, Indianapolis, Indiana; 6 Phillip M. Drayer Department of Electrical and Computer Engineering, Lamar University, Beaumont, Texas; 7 Translational Informatics, Biostatistics and Epidemiology Lab, Indiana University School of Medicine, Indianapolis, Indiana; 8 Department of Pediatrics, Indiana University School of Medicine, Indianapolis; 9 Department of Family Medicine, Indiana University School of Medicine, Indianapolis, Indiana; 10 Comprehensive Center for Brain Health, Department of Neurology, University of Miami Miller School of Medicine, Boca Raton, Florida

## Abstract

**Importance:**

Detection of Alzheimer disease and related dementias (ADRD) is a challenge in primary care settings. The Quick Dementia Rating System (QDRS) and the Passive Digital Marker (PDM) were developed as 2 scalable tools for early detection of ADRD. The QDRS is a patient-reported outcome measure, while the PDM is a machine learning algorithm that uses electronic health record (EHR) data. Both can be embedded in the EHR for ease of use.

**Objective:**

To evaluate the effect of the combined approach of QDRS plus PDM in improving the annual rate of new documented ADRD diagnoses in primary care practices.

**Design, Setting, and Participants:**

This randomized clinical trial was performed between July 2, 2022, and July 1, 2024. Nine federally qualified primary health care clinics in Indianapolis, Indiana, were randomized to usual care (no routine screening for ADRD), PDM only, or QDRS plus PDM. Participants were treated as per the randomization arm. Participants included adults 65 years and older, without a diagnosis of mild cognitive impairment, dementia, or severe mental illness. Data analysis was based on intention to treat and was performed between November 1, 2024, and August 20, 2025.

**Intervention:**

Randomization of clinic to usual care, PDM only, or QDRS plus PDM.

**Main Outcomes and Measures:**

The primary outcome was 12-month cumulative incidence of ADRD diagnoses; the secondary outcome was any ADRD diagnostic workup, such as laboratory tests, neuropsychological testing, or brain imaging.

**Results:**

The study included 5325 patients (mean [SD] age, 71.1 [5.9] years), 3312 (62.2%) of whom were female. Compared with the usual care clinics (12-month incidence, 213 of 1724 [12.4%]), the odds of an incident ADRD diagnosis were higher in the clinics randomized to QDRS plus PDM (12-month incidence, 355 of 2301 [15.4%]; adjusted odds ratio [AOR], 1.31; 95% CI, 1.05-1.64) but not the clinics randomized to PDM only (12-month incidence, 134 of 1300 [10.3%]; AOR, 0.84; 95% CI, 0.63-1.11]). Compared with the usual care clinics (12-month incidence, 500 of 1724 [29.0%]), the odds of ADRD diagnostic assessments were higher in the QDRS plus PDM clinics (12-month incidence, 844 of 2301 [36.7%]; AOR, 1.41; 95% CI, 1.12-1.77) but not the PDM clinics (12-month incidence, 362 of 1300 [27.8%]; AOR, 0.94; 95% CI, 0.72-1.22).

**Conclusions and Relevance:**

This randomized clinical trial found that the combined approach was effective at scale for the early detection of ADRD in primary care settings. This is an important feature in busy primary care settings that can benefit both the health care system and patients.

**Trial Registration:**

ClinicalTrials.gov Identifier: NCT05231954

## Key Points

**Question:**

Compared with usual care, what is the effect of a combined approach using the Quick Dementia Rating System (QDRS), a patient-reported outcome tool, and a Passive Digital Marker (PDM), a machine learning algorithm that uses existing data captured by the electronic health record, in improving the annual rate of a new documented Alzheimer disease and other related dementia (ADRD) diagnosis in federally qualified health centers?

**Findings:**

This randomized clinical trial including 5325 patients found that the odds of new ADRD diagnoses was 31% higher in the clinics randomized to the combined QDRS and PDM approach compared with usual care clinics.

**Meaning:**

A scalable, cost-effective approach for early detection of ADRD is possible with limited additional time and effort from the primary care team in federally qualified health centers.

## Teaser

This randomized clinical trial evaluates the effectiveness of an approach combining the Quick Dementia Rating System and Passive Digital Marker machine learning algorithm for the detection of Alzheimer disease and other related dementias in primary care.

## Introduction

Three recent breakthroughs are reshaping primary care services provided for people living with Alzheimer disease and related dementias (ADRD). First, lifestyle modifications can reduce the risk of ADRD by as much as 45%.^1^ Second, therapies to lower amyloid levels for early stages of Alzheimer disease are now available.^2^ Third, the Center for Medicare and Medicaid Innovation has adopted a comprehensive care model to support individuals living with ADRD and their families.^3^ However, more than 50% of older adults in primary care never receive a formal and timely diagnosis.^3,4,5,6^ Thus, the National Plan to Address Alzheimer Disease identified ADRD detection as a core aim for improving the quality of care for older adults.^7^

Using digital or paper versions of cognitive performance tests administered by clinicians have scalability and sustainability challenges in primary care.^4,8,9,10,11,12,13,14,15,16,17,18,19^ The recent US Food and Drug Administration approval of a blood-based biomarker is promising for the detection of Alzheimer disease; however, biomarkers that can identify other ADRD pathologies are lacking. Patient-reported outcome (PRO) approaches, the growth in data captured by various electronic health record (EHR) systems, and the advances in machine learning algorithms may overcome such challenges while avoiding additional time requirement from clinicians for data collection.^19,20,21,22^ The Quick Dementia Rating System (QDRS) was initially developed as an informant rating instrument to detect and stage ADRD^19^ and was subsequently revised and externally validated as a PRO tool.^23,24,25,26,27^ This version of the QDRS consists of 10 multiple choice questions that capture cognitive, functional, behavioral, and psychological outcomes reported by the patient in less than 3 minutes with an accuracy of 85% for ADRD diagnosis.^23,24,25,26,27^ Together with other investigators, members of our group also developed and externally validated the Passive Digital Marker (PDM) that uses a machine learning algorithm to extract data from the EHR for early detection of ADRD with 80% accuracy.^28,29,30^ We conducted a randomized clinical trial (RCT) to evaluate the effectiveness of a combined QDRS and PDM approach for the detection of ADRD in primary care. Our primary hypothesis was that compared with usual care (with no routine screening for ADRD), the combined approach (QDRS plus PDM) would increase the incidence rate of a documented ADRD diagnosis from 6% to 13%.^11,12^ Our secondary hypothesis was that compared with usual care, the combined approach would have higher rates for ADRD diagnostic workup following a positive screen result from 44% to 66%. We further hypothesized that the combined approach would overcome the low implementation rate of cognitive screening in primary care, including the completion rate of PRO tools.^11,18^ This approach benefits from directly identifying patterns of health from the EHR indicative of early ADRD while mitigating known limitations of EHR data with important PRO-reported information.

## Methods

This RCT was approved by the institutional review board of Indiana University at Indianapolis. A waiver of informed consent was granted for the use of patient EHR data to determine eligibility, study enrollment, and study outcomes. The published protocol^31^ appears in Supplement 1, and the study followed the Consolidated Standards of Reporting Trials (CONSORT) reporting guideline.

### Clinical Setting

The trial was conducted in 9 urban federally qualified health centers affiliated with Eskenazi Health in Indianapolis. Eskenazi Health is one of the largest safety-net integrated health care systems in the nation. Each of these primary care sites is geographically located in high-need, low-income, and diverse neighborhoods that require interpretation services. We randomized clinics to 1 of 3 approaches at a 1:1:1 ratio: continue with usual care (control), use of PDM alone, and combined use of the QDRS plus PDM. A computer-generated randomization scheme was used for the random assignment of the clinics. The clinic-level randomization was chosen for practical reasons and to prevent contamination by preference of patient or physician (selection bias). Patients were eligible if they were 65 years or older and had an established primary care clinician with at least 3 years of electronic health data. Patients were excluded if they had a documented diagnosis of ADRD or mild cognitive impairment (MCI) (identified via *International Statistical Classification of Diseases, Tenth Revision* [*ICD-10*] codes), had a history of cholinesterase inhibitor or memantine use, had a diagnosis of a serious mental illness, or resided in a long-term care facility. All eligible patients who visited any of the study clinics during the study period were included in our analysis.

### Interventions

We embedded the QDRS and PDM into the EHR as 2 scalable and externally validated tools for early detection of ADRD.^23,24,25,28,29,30^ The QDRS has an overall accuracy of 80% at a cutoff threshold of 1.5 with a sensitivity of 88% and specificity of 76%.^23^ The PDM has an overall accuracy of 85% at a cutoff probability threshold of 0.5 with a sensitivity of 76% and specificity of 80%.^28^ The cutoff score for PDM was a risk score of greater than 59% and, for the QDRS, a total score of greater than 1.5 (range, 0-30, with higher scores indicating cognitive impairment). Eskenazi Health used an EHR system (EPIC Systems Corporation) with a clinical decision support (CDS) engine that included a customizable patient-facing survey platform used to administer the QDRS (eAppendix in Supplement 2). The EHR contained detailed clinical data that were accessed to run the PDM algorithm. Patient registration at the primary care clinic triggered the CDS process, including an invitation to the patient to complete the QDRS via a patient portal (MyCHART; EPIC Systems Corporation) for those clinics randomized to QDRS plus PDM. The PDM or the QDRS results (if positive) were displayed in a secure EHR user interface via a direct inbox message to a clinician with emphasis on key features of the PDM or the QDRS that explain an individual patient’s positive result (eFigure 3 in Supplement 2). PDM results were only shared with clinicians practicing in the clinics randomized to either PDM or QDRS plus PDM. We monitored the uptake and use of the CDS engine using the system logs. The uptake during the go-live period appeared problematic; thus, we were able to revisit training and socializing the new workflow with clinical staff and alter the user interface based on feedback. The final CDS alerts recommended a referral to the Healthy Aging Brain Care Center at Eskenazi Health for any patient with a positive screen result. The diagnostic assessment conducted by the Healthy Aging Brain Care Center includes a structured informant interview, physical and neurological examination of the patient, and completion of neuropsychological testing, brain imaging, and laboratory tests for thyrotropin, vitamin B_12_, or folate levels or syphilis.^32^

### Data Collection

We collected deidentified EHR data for patients receiving care in all the clinics. Race and ethnicity were classified by the EHR via self-reporting from the patient. They were assessed to address health disparities among underserved communities. Race was categorized as Black or African American, White, or other race (including American Indian or Alaska Native, Asian, Native Hawaiian or Other Pacific Islander, and multiracial); ethnicity was categorized as Hispanic or Latino and non-Hispanic or non-Latino.

The primary outcome measure was any new (incident) ADRD case identified (documented in the EHR via *ICD-10* codes for ADRD or MCI) within 12 months of the index visit (ie, the first primary care clinic visit where an eligible patient is seen). Eligible patients across all clinics have completed the PDM algorithm, even though the results of the PDM was shared only with the clinicians at the PDM or the QDRS plus PDM clinics. Data from the previous dementia screening trial by Fowler et al^12^ and a recently published cognitive screening trial^33^ indicated that all diagnostic workup services after a positive screen would occur within the first 90 days of the index visit (ie, the first clinic visit where an eligible patient is seen by a primary care clinician after the rollout of the PDM at Eskenazi Health). We extended the follow-up period in the present study to 12 months to accommodate any logistical delay related to the completion of imaging tests and visits to memory care clinics. The secondary outcome measure consisted of cognitive diagnostic assessments that clinicians may have ordered to diagnose or exclude ADRD such as laboratory tests for thyrotropin, serum vitamin B_12_, or folate levels or syphilis at any point during the 90 days after the index date; neuropsychological testing during the 12 months after the index date; imaging of the head and neck, brain, or skull during the 12 months after the index date; and medications approved for management of ADRD (ie, cholinesterase inhibitors, memantine) during the 12 months after the index date.

### Statistical Analysis

Statistical analysis was performed between November 1, 2024, and August 20, 2025. All data analyses were conducted based on the intention-to-treat protocol. Our primary analysis was conducted at the individual patient level.

At the individual patient level, we evaluated the effect of screening approaches (targeted at clinics) on the odds of a patient receiving an incident ADRD diagnosis (primary outcome) or related diagnostic assessments (secondary outcome). Generalized linear mixed models (GLMM) were used to determine intervention effectiveness on these patient-level outcomes. GLMM models included fixed effects for patient (level 1: age, sex, race, and ethnicity) and clinic (level 2: study intervention arm) factors and a random intercept to model patients nested within clinics to account for potential variability of care across clinics. Patient characteristics such as sex, race, ethnicity, and age at enrollment were adjusted for in our final models as potential confounders to generate conditional odds ratios (ORs) as a measure of intervention effect. GLMM models included degrees-of-freedom corrections based on the between-within heuristic^34^ to maintain appropriate type I error due to the small number of clusters (ie, 9 clinics).

#### Sensitivity Analysis

At the clinic level, we assessed the robustness of intervention effectiveness under the assumption that variance in how patients were managed would be partly explained at the clinic level.^34,35,36^ Our cluster-level analysis involved a 2-step process: (1) summarize data at the cluster level, that is, the cumulative incidence proportion of outcome (ADRD diagnosis, ADRD diagnostic services) within a cluster, and (2) compare cluster-level means between study arms using a weighted linear regression (similar to a weighted 1-way analysis of variance) with a Tukey adjustment to control for inflation of family-wise error rate due to multiple group (between study arm) comparisons. The numbers of patients in clinics were used as weights.

#### Additional Analysis

We used cumulative incidence plots to characterize the time to an incident ADRD diagnosis (primary outcome). A Gray test was used to examine whether a patient’s time to an incident ADRD diagnosis or diagnostic-related assessment differed between study arms. A mixed-effects Cox proportional hazards regression model with shared frailty (random intercept for clinics) was used to estimate intervention effect on time to an ADRD diagnosis. The outcome variable was defined as the time from a patient’s index visit to an incident ADRD diagnosis or the end of study (365 days or 12 months after index visit), whichever came first. Patients were right censored if the study end date was reached before an ADRD diagnosis was documented. Cox proportional hazards regression models included adjustment for sex, race, ethnicity, and age at enrollment as potential confounders. Receipt of any services for cognitive diagnostic assessment was analyzed as a time-varying covariate to determine whether time to an ADRD diagnosis after any services for cognitive diagnostic assessment differed by study arm. Hazard ratios (HRs) with 95% CIs were used to summarize intervention effect. Similar methods (ie, cumulative incidence plots and mixed-effects Cox proportional hazards regression models) were used to assess intervention effect on time to any services for cognitive diagnostic assessment (secondary outcome). In an exploratory analysis, we used GLMM models to examine whether having a positive screening result was associated with higher odds (or a cumulative incidence) of ADRD diagnoses in PDM and QDRS plus PDM study arm clinics than the control arm clinics.

All analyses were performed in R studio, version 4.1.3 (R Program for Statistical Computing), using the glmer, tidycmprsk, and coxme packages. Two-sided *P* < .05 indicated statistical significance.

## Results

**Figure 1.**
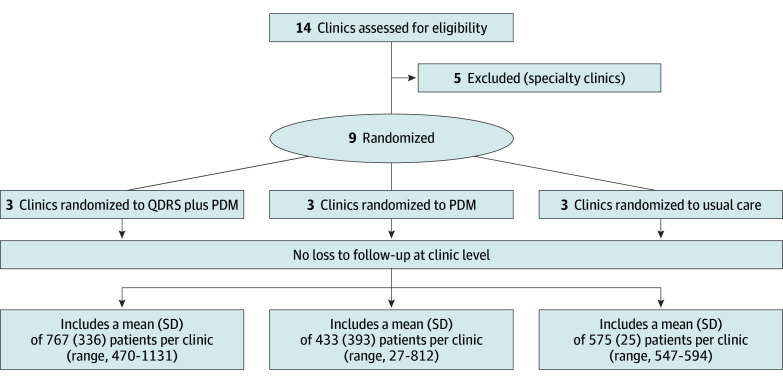
Flow Diagram of Study Clinics and Participants PDM indicates Passive Digital Marker; QDRS, Quick Dementia Rating System.

Of 14 clinics available for study recruitment, 9 were eligible and agreed to study participation. The number of eligible patients per clinic varied between and within study arms with a range from 27 to 1131 (Figure 1 and Table 1). The 9 clinics had a total of 5325 eligible patients 65 years and older with a mean (SD) age of 71.1 (5.9) years. A total of 3312 patients (62.2%) were female and 2013 (37.8%) were male. In terms of race, 2927 patients (55.0%) were Black or African American, 1789 (33.6%) were White, and 609 (11.4%) were of other race or ethnicity; in terms of ethnicity, 826 patients (15.5%) were Hispanic or Latino and 4499 (84.5%) were non-Hispanic or non-Latino.

**Table 1.** Participant Characteristics by Study Clinic Randomization

Characteristic	Clinic group		
	Control (n = 1724)	PDM (n = 1300)	QDRS plus PDM (n = 2301)
Individual level			
Sex, No. (%)			
Female	1036 (60.1)	827 (63.6)	1449 (63.0)
Male	688 (39.9)	473 (36.4)	852 (37.0)
Race, No. (%)			
Black or African American	986 (57.2)	605 (46.5)	1336 (58.1)
White	575 (33.4)	471 (36.2)	743 (32.3)
Other^a^	163 (9.5)	224 (17.2)	222 (9.6)
Ethnicity, No. (%)			
Hispanic or Latino	222 (12.9)	339 (26.1)	265 (11.5)
Non-Hispanic or non-Latino	1502 (87.1)	961 (73.9)	2036 (88.5)
Age at start of study, y			
Mean (SD)	71 (6)	71 (6)	71 (6)
Median (IQR) [range]	70 (67-74) [64-102]	69 (66-74) [64-102]	69 (67-74) [64-98]
Cluster level			
No. of clinics	3	3	3
No. patients per clinic			
Mean (SD)	575 (25)	433 (393)	767 (336)
Median [range]	583 [547-594]	461 [27-812]	700 [470-1131]

Abbreviations: PDM, Passive Digital Marker; QDRS, Quick Dementia Rating System.

^a^ Includes American Indian or Alaska Native (17 [0.10%]), Asian (38 [0.7%]), Native Hawaiian or Other Pacific Islander (31 [0.6%]), and multiracial (523 [9.8%]).

### Intervention Effect on Incidence of ADRD Diagnoses

Overall, after 12 months of follow-up from the index visit, 355 of 2301 patients (15.4%) in QDRS plus PDM clinics had received a diagnosis compared with 134 of 1300 (10.3%) in PDM clinics and 213 of 1724 (12.4%) in usual care clinics. Compared with the usual care clinics, the odds of an incident ADRD diagnosis were 31% higher in the combined QDRS plus PDM clinics (adjusted OR [AOR], 1.31 (95% CI, 1.05-1.64) but not the PDM-only clinics (AOR, 0.84; 95% CI, 0.63-1.11) (Table 2). Less than 1% of the variation in the incidence of ADRD was explained by clinic differences or differences among patients in the same clinic as demonstrated by an intracluster correlation coefficient of less than 0.01.

**Table 2.** Generalized Linear Mixed-Effects Model Results: Intervention Effect on ADRD Diagnosis and Diagnostic Assessments During a 12-Month Follow-Up^a^

Outcome	Control, IR (%)	Effect by clinic group									
		PDM					QDRS + PDM				
		IR (%)	Crude OR (95% CI)	*P* value	Adjusted OR (95% CI)^b^	*P* value	IR (%)	Crude OR (95% CI)	*P* value	Adjusted OR (95% CI)^b^	*P* value
Primary: ADRD diagnosis	213 (12.4)	134 (10.3)	0.82 (0.62-1.07)	.08	0.84 (0.63-1.11)	.13	355 (15.4)	1.29 (1.04-1.61)	.006	1.31 (1.05-1.64)	.004
Secondary: ADRD diagnostic assessments	500 (29.0)	362 (27.8)	0.95 (0.73-1.25)	.67	0.94 (0.72-1.22)	.58	844 (36.7)	1.41 (1.11-1.79)	<.001	1.41 (1.12-1.77)	<.001

Abbreviations: ADRD, Alzheimer disease and related dementia; IR, 12-month cumulative incidence rate; OR, odds ratio; PDM, Passive Digital Marker; QDRS, Quick Dementia Rating System.

^a^ Generalized linear mixed-effects models include a between-within correction to maintain appropriate type I error.

^b^ Includes covariate adjustment for differences in age, sex, race, and ethnicity distribution.

**Figure 2.**
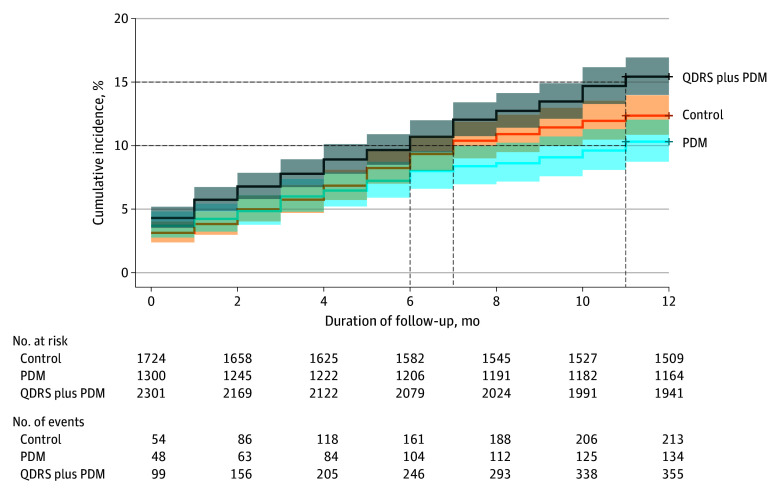
Cumulative Incidence of Alzheimer Disease and Related Dementias Diagnosis by Study Arm During a 12-Month Follow-Up Period PDM indicates Passive Digital Marker; QDRS, Quick Dementia Rating System.

ADRD diagnoses occurred earlier in the QDRS plus PDM clinics than the usual care clinics (Figure 2). Independent of patient characteristics (sex, age, race, and ethnicity), the Cox proportional hazards regression model results showed that the odds of earlier diagnoses were higher in combined clinics with an adjusted hazard ratio (AHR) of 1.37 (95% CI, 1.12-1.68) than in the usual care clinics (Table 3). Moreover, the odds of an earlier ADRD diagnosis were 2 times higher after the receipt of any services for cognitive diagnostic assessment (AHR, 2.07; 95% CI, 1.77-2.42).

**Table 3.** Cox Mixed-Effects Model Results: Intervention Effect on Time to ADRD Diagnosis and Diagnostic Assessments During a 12-Month Follow-Up

Outcome	Effect by clinic group							
	PDM				QDRS plus PDM			
	Crude HR (95% CI)	*P* value	Adjusted HR (95% CI)^a^	*P* value	Crude HR (95% CI)	*P* value	Adjusted HR (95% CI)^a^	*P* value
Primary: ADRD diagnosis	1.08 (0.83-1.40)	.58	1.14 (0.88-1.49)	.32	1.43 (1.16-1.75)	<.001	1.37 (1.12-1.68)	.002
Secondary: ADRD diagnostic assessments	0.95 (0.81-1.11)	.50	0.93 (0.80-1.10)	.41	1.32 (1.15-1.50)	<.001	1.30 (1.14-1.49)	<.001

Abbreviations: ADRD, Alzheimer disease and related dementia; HR, hazard ratio; PDM, passive digital marker; QDRS, Quick Dementia Rating System.

^a^ Includes covariate adjustment for differences in age, sex, and race distribution.

### Intervention Effect on Incidence of ADRD Diagnostic Assessments

After a 12-month of follow-up from the index visit, 844 of 2301 patients (36.7%) in the QDRS plus PDM clinics had completed a ADRD-related diagnostic assessment compared with 362 of 1300 (27.8%) in PDM clinics and 500 of 1724 (29.0%) in usual care clinics. Overall, the Cox proportional hazards regression model results showed that the odds of earlier services for diagnostic assessments were 30% higher in the combined clinics (AHR, 1.30; 95% CI, 1.14-1.49) compared with usual care clinics (Table 3 and eFigure 1 in Supplement 2). Patients in the combined clinics had an earlier time to laboratory tests (assessments) than usual care clinics (eFigure 2 in Supplement 2). Overall, compared with the usual care clinics, the odds of ADRD diagnostic assessments were higher in the QDRS plus PDM clinics (AOR, 1.41; 95% CI, 1.12-1.77) but not the PDM clinics (AOR, 0.94; 95% CI, 0.72-1.22) (Table 2). Our sensitivity analyses using a weighted linear regression model corroborated GLMM results by showing a higher mean cumulative incidence of ADRD diagnostic assessments and diagnoses in the combined clinics than the usual care clinics (eTable 1 in Supplement 1).

### Mechanism of Intervention Effect on Incidence of ADRD Diagnoses 

Explanatory analyses were implemented to examine why the QDRS plus PDM clinics were associated with higher odds of ADRD diagnoses than the usual care clinics. Our GLMM and mixed-effects Cox proportional hazards regression model results showed that irrespective of study arm, patients who had a positive PDM screening result had higher and earlier odds (cumulative incidence) of an incident ADRD diagnosis (eTables 2 and 3 in Supplement 1). Even with missing QDRS results in the combined study arm, patients with a positive PDM screen result had a higher cumulative incidence of diagnostic assessments (275 of 663 [41.5%] vs 400 of 1162 [34.4%]) and diagnoses (159 of 663 [24.0%] vs 131 of 1162 [11.3%]) than those with a negative PDM result. Similarly, despite having a low completion rate for the QDRS in the combined study arm (476 of 2301 [20.7%]), having a positive QDRS result (irrespective of PDM results) was also associated with higher odds of an incident ADRD diagnosis. Overall, the higher incidence of ADRD diagnoses among patients with a positive (vs negative) PDM or QDRS screen result is corroborated by higher and earlier odds (cumulative incidence) of ADRD diagnostic assessments (eTables 2 and 3 in Supplement 2).

## Discussion

In this RCT, we found that using the QDRS coupled with the PDM could represent a very effective approach for the detection of ADRD by nudging the primary care clinicians to complete diagnostic cognitive assessments following a positive screen result. Commonly available approaches to ADRD detection in primary care face a major barrier in that they depend on clinicians performing the data collection (eg, through direct interview or testing).^4,6,8^ We eliminated this barrier by combining a PRO tool, the QDRS, with an EHR-based early detection machine learning risk classifier, the PDM.

There is a paucity of RCTs that evaluate the effect of screening for ADRD on the incidence rates of ADRD diagnosis in primary care settings. Moreover, the few studies that exist have mixed results for the effectiveness of ADRD screening interventions on increasing diagnostic workup and subsequent diagnoses among at-risk patients.^33,37^ Based on a comparable study population, an RCT conducted in Indiana^12^ showed that compared with usual care, a brief cognitive assessment (Memory Impairment Screen or the Mini Cog in person or via the telephone) completed by a research assistant did not increase documented ADRD diagnosis (3.4% vs 3.1%; *P* = .70). Conversely, another RCT conducted in the Bronx, New York, among patients with cognitive complaints^33^ showed an OR of 3.43 (95% CI, 2.32-5.07) for improvement in ADRD care (ie, any documentation in the EHR of new diagnosis of MCI or ADRD, laboratory or imaging tests, new medication prescription for ADRD, or specialist referral for ADRD evaluation) when patients were randomized to receive a brief cognitive assessment vs usual care. Together these mixed results from screening interventions on increasing the rate of incident ADRD diagnoses may be due to differences in the target population (asymptomatic vs symptomatic), the types of nudges used to change the behaviors of the clinicians (letter or token vs electronic alerts), or outcome measures (incidence rate of new diagnosis vs composite scores of ADRD care). Consistent with the Bronx RCT, our study results show that electronic alerts can effectively change clinician behavior by increasing the incidence of ADRD diagnostic assessments that result in earlier detection of ADRD.

### Limitations

This study has some limitations. First, the QDRS completion rate was low. Given that the PDM alone was not effective, it is notable that the combined QDRS plus PDM approach seems more impactful than would be expected with such a low QDRS completion rate. That said, this finding is important because it has a strong implication on the broader applicability of PROs for ADRD detection. Moreover, we hypothesize that for the QDRS plus PDM clinics, the QDRS may have increased the trust of the clinician in the results of the PDM.^38^ Trust in machine learning is influenced by a combination of human factors, such as user education, past experiences, user biases, and perception toward automation.^39^ The attenuated effect of the PDM alone signals a precautionary lesson on the limits of using a PDM to detect ADRD. Second, our study did not randomize clinics into a QDRS only arm. Thus, we do not know if the combined QDRS plus PDM approach is better than using the QDRS only. Third, this work involves a single health care system serving socially and medically underserved populations, which may limit generalizability. During the past 3 years, 25 156 patients 65 years and older had at least 1 completed clinical encounter in Eskenazi Health. Among those with completed encounters, 10 059 patients (40.0%) had an active a patient portal. In a recent study conducted at the same federally qualified health center, the mean (SD) educational level of older primary care patients who underwent a comprehensive brain health assessment was 13.1 (2.6) years.^5^ Thus, the completion of the QDRS in the present study may be due to digital dexterity barriers related to access the patient portal, the need for language translation, and the relatively low educational level of older adults served by the enrolled primary care clinics. Fourth, the precision of our data collection concerning the presence of cognitive diagnostic follow-up is biased toward high false-positive rates due to the imprecise nature of using EHRs. Some of the selected brain imaging and blood tests, such as thyrotropin, vitamin B_12_, and folate levels, may be ordered for reasons unrelated to cognition. However, the randomization process would distribute these false-positive results across the 3 arms. Last, although our study indicated that the combined approach for early detection led to a 31% increase in the rate of documented ADRD, the most recent review by the US Preventive Services Task Force found insufficient evidence to recommend for or against screening for cognitive impairment in primary care.^4^

## Conclusions

This RCT found that a combined scalable approach using PROs coupled with a PDM increased the incidence rate of ADRD diagnosis within 12 months by one-third. Moreover, the combined approach did not require additional time, labor, or effort from the clinical team. The scalability feature of combining the QDRS and the PDM could be replicated using other tools that use the passive data processes of the PDM and the QDRS. This is an important feature in busy primary care settings, which benefits both the health care system and patients.

## Notes

Supplement 1.
Trial Protocol

Supplement 2. **eFigure 1.** Cumulative Incidence of Completed ADRD Diagnostic Assessments by Study Arm During a 12-Month Follow-Up **eFigure 2.** Cumulative Incidence of Completed ADRD Diagnostic Assessments by Study Arm During a 12-Month Follow-Up Period by Type of Diagnostic Assessment **eAppendix.** The Computerized Decision Support Workflow and Display **eFigure 3.** The In-Basket Message to the Primary Care Clinician **eTable 1.** Intervention Effect on the Cumulative Incidence of ADRD Diagnosis and Diagnostic Assessments at the Clinic Level **eTable 2.** Generalized Linear Mixed Model Results: Intervention Effect (by Screening Results) on ADRD Diagnosis and Diagnostic Assessments During a 12-Month Follow-Up **eTable 3.** Mixed-Effects Cox Model Results: Intervention Effect (by Screening Results) on Time to ADRD Diagnosis and Diagnostic Assessments During a 12-Month Follow-Up

Supplement 3.
Data Sharing Statement
