# Supplementary material for: Digital Detection of Dementia in Primary Care: A Randomized Clinical Trial
Source: JAMA Netw Open. 2025 Nov 10;8(11):e2542222. doi: 10.1001/jamanetworkopen.2025.42222 (PMC12603861; doi:10.1001/jamanetworkopen.2025.42222)
Supplement: Supplement 1. — Trial Protocol [file jamanetwopen-e2542222-s001.pdf]

Digital Detection of Dementia (D^3) Studies

Malaz Boustani, MD, MPH
410 West 10th Street, Suite 1140, Indianapolis, IN 46202

Zina Ben-Miled, PhD
723 W Michigan St, SL 160, Indianapolis, IN 46202

James Galvin, MD
777 Glades Rd, BC-71 Room 209, Boca Raton, FL 33431

Nicole Fowler, PhD, Associate Professor of Medicine
General Internal Medicine and Geriatrics
Indiana University

Paul Dexter, MD, Associate Professor of Clinical Medicine
General Internal Medicine and Geriatrics
Indiana University

Randall Grout, MD, Assistant Professor of Pediatrics
Pediatrics
Indiana University

Patrick Monahan, PhD, Professor of Biostatistics
Biostatistics
Indiana University

Michael Kleiman, PhD, Data Scientist
School of Medicine
University of Miami

Support Provided by:
National Institutes of Health
R01AG069765-01

Table of Contents

Abbreviations ………………………………………………………………. 3
Specific Aims ………………………………………………………………. 4
A. Significance ………………………………………………………… 6
B. Innovation …………………………………………………………… 8
C. Preliminary Work ……………………………………………………. 9
D. Research Approach …………………………………………….......... 14
E. Protection of Human Subjects ………….………………………… 22
F. Data and Safety Monitoring Plan …………………..……………. 25
References …………………………………………………………….….. 31

Abbreviations

ADRD	Alzheimer’s Disease and related dementias
AE	Adverse Event
AUC	Area Under the Curve
AWV	Annual Wellness Visit
CDR	Clinical Dementia Rating
CDS	Computerized decision support
CV	Coefficient of variation
DHHS	Department of Health and Human Services
DSMB	Data Safety Monitoring Board
DRE	Disease-Related Event
Dx	Diagnosis
EHR	Electronic health record
GCP	Good Clinical Practice
GLP	Good Laboratory Practices
GMP	Good Manufacturing Practices
GPI	Generic Product Identifier
HIPAA	Health Insurance Portability and Accountability Act
ICC	Intraclass correlation coefficients
IF	Inflation factor
IRB	Institutional Review Board
ISM	Independent Safety Monitor
ISO	International Organization for Standardization
IUPUI	Indiana University-Purdue University-Indianapolis
MCI	Mild Cognitive Impairment
ML	Machine learning
NCI	No Cognitive Impairment
NCT	National Clinical Trial
NIA	National Institute on Aging
NIH	National Institutes of Health
NPI	Neuropsychiatric Inventory
Nx	Medical notes
OHRP	Office for Human Research Protections
PDM	Passive Digital Marker
PI	Principal Investigator
PRO	Patient-reported outcome
QA	Quality Assurance
QC	Quality Control
QDRS	Quick Dementia Rating Scale
Rx	Prescriptions
SAE	Serious Adverse Event
SCI	Subjective Cognitive Impairment
SOP	Standard Operating Procedure
TSH	Thyroid-stimulating hormone
UDS	Uniform Data Set
UHealth	University of Miami
UP	Unanticipated Problem
US	United States

SPECIFIC AIMS

Alzheimer's disease and related dementias (ADRD) negatively impact millions of Americans with an annual societal cost of more than $200 million.[1] Currently, half of Americans living with ADRD never receive a diagnosis.[2-7] For those who do, the diagnosis often occurs two to five years after the onset of symptoms.[6-9] As stated by the National Institute on Aging (NIA) (RFA-AG-20-051) "*The inability to diagnose and treat cognitive impairment results in prolonged and expensive medical care*" and "*early detection could help persons with dementia and their care partners plan for the future*". Furthermore, if the development of disease modifying therapeutics for ADRD is successful, this may require the use of such therapeutics at a very early stage of ADRD.[1] However, the current approaches of using cognitive tests or biomarkers for early detection of ADRD are not scalable due to their low acceptance, their invasive nature, their cost, or their lack of accessibility in rural or underserved areas. Thus, the NIA called out for the development of low cost, effective, and scalable approaches for early detection of ADRD (RFA-AG-20-051).

Over the past five years, two interdisciplinary scientific teams led by Drs. Boustani, Ben-Miled, and Galvin (MPI on this proposal) have developed and tested scalable approaches for the early detection of ADRD. At Indiana University, Drs. Boustani and Ben-Miled leveraged the widely available electronic health record (EHR) data and advances in Machine Learning algorithms to develop a Passive Digital Marker (PDM) for early detection of ADRD with 80% accuracy for one-year and three-year prediction horizons.[10] At the same time, Dr. Galvin's team developed and tested the Quick Dementia Rating Scale (QDRS) as a practical 2-3 minute patient-reported outcome (PRO) tool, for both early detection and staging of ADRD with 85% accuracy for ADRD diagnosis.[11] If implemented successfully within the current Medicare funded Annual-Wellness Visit (AWV),[2] an integrated approach based on the above two ADRD detection approaches (the PDM and the QDRS) may overcome the current barriers to early ADRD detection in a cost- and time-efficient manner that can be generalized across any primary care practice.

In response to the RFA-AG-20-051 call for the "validation, and translation of screening and assessment tools for measuring cognitive decline," our interdisciplinary scientific team is proposing to execute a clinical validation study, one pragmatic trial and one additional replicated pragmatic trial among patients from diverse rural, suburban and urban primary care practices in two independent sites located in central Indiana and south Florida. As required by the RFA, the first study will be a single-arm clinical validation (NIH Stage III) study of our developed early ADRD detection approaches; Passive Digital Marker (PDM) and the Patient Reported Outcome (PRO) tool, the Quick Dementia Rating System (QDRS) for discriminating gold standard ADRD diagnoses for a sample of 400 older primary care patients served in the two states. The other studies will be two pragmatic cluster-randomized controlled comparative effectiveness (NIH Stage IV) trials to incorporate the PDM and the QDRS within the Medicare paid Annual Wellness Visit (AWV) for a cohort of patients from practices across the two independent sites, with practices randomized in each pragmatic trial to one of the 3 arms (the AWV alone, the AWV with PDM, and the AWV with both the PDM and the QDRS).

These studies will support the following aims:

Primary Aim 1: Evaluate the predictive performance of the PDM, the QDRS, and the combined (PDM + QDRS) approach in the early detection of ADRD, compared to the gold standard diagnoses.

Primary Hypothesis 1: The Area Under the Curve (AUC) for the combined approach (PDM + QRDS) will be 0.85 or greater and the AUC for the PDM or the QDRS will be 0.75 or greater.

Secondary Hypothesis 2: The combined approach (PDM + QDRS) will have statistically better AUC than each individual approach alone.

Primary Aim 2: Evaluate the practical utility and effect of the PDM, the QDRS, and the combined approach (PDM + QDRS) in improving the annual rate of new documented ADRD diagnosis in primary care practices.

Primary Hypothesis 1: In comparison to the AWV alone, the combined approach (PDM + QDRS) within the AWV will increase the incidence rate of ADRD over the subsequent 12 months from 6% to 13%.

Secondary Hypothesis 2: In comparison to the AWV alone, the combined approach (PDM + QDRS) within the AWV will have higher acceptance rates for recommended ADRD diagnostic work-up following a positive screen from 44% to 66%.

A. SIGNIFICANCE: Our proposal is designed to help clarify the following important questions.

A.1. What is the value of early detection of Alzheimer Disease and Related Dementias (ADRD)? Primary care clinicians provide the vast majority of care to older adults living with ADRD.[1-5] However, more than 50% of these older adults never receive a formal diagnosis.[4-9, 12] And for those patients who are diagnosed with ADRD, the diagnosis often occurs 2-5 years after symptom onset when the syndromes reach mild to moderate stage.[4-9] The inability to diagnose ADRD and manage its cognitive, functional, and psychological disabilities results in significant burden for patients, families, and the entire society.[1, 14] Early detection of ADRD could reduce this burden.[13,15-17] Furthermore, early detection will become increasingly important for ensuring that people receive early ADRD disease modification treatment when available.[1] Thus, the National Academy of Sciences, National Plan to Address Alzheimer's Disease, and Affordable Care Act all describe earlier ADRD detection as a core aim for improving the quality of care for older adults.[1,2,20]

A.2. Can the Annual Wellness visit (AWV) overcome barriers for early detection of ADRD? Beginning in 2011, the Centers for Medicare and Medicaid Services (CMS) started paying providers (with no patient requirement for copayment) for the AWV.[2,18] Although the AWV does not mandate formal ADRD screening, the beneficiary's cognitive function is assessed through direct observation, potentially supplemented with information provided by family members. In 2018, we published the results of a study that evaluated the ability of the current format of the AWV to improve ADRD detection.[19] Using de-identified administrative claims data for a nationally representative sample of Medicare beneficiaries, we found no impact for the AWV in its current format on the diagnostic rate, testing, or care management for ADRD.[19] Enhancing the AWV with integrated scalable formal screening will likely facilitate earlier identification of ADRD.[1,2]

A.3. Can we leverage Electronic Health Record (EHR) systems and Machine Learning (ML) algorithms for the development of a low-cost approach for early detection of ADRD and embed such an approach within the AWV? Growth in data captured by various EHR systems, increased access to inexpensive computational power and advancements in ML algorithms offer opportunities to answer the NIA call and develop targeted and scalable approaches for the early detection of ADRD. These approaches may allow feasible and scalable segmentation of patient populations including a high-risk group that could be targeted for further invasive, expensive, and time consuming cognitive or biological screening tests. However, one of the most difficult aspects of working with EHR data is its heterogeneous nature, with many different data types (e.g., continuous versus categorical, structured versus unstructured) and the common state of missing values for any number of variables per patient, as not all tests are administered to each patient or recorded correctly if they are. While such heterogeneity makes it difficult to apply various ML algorithms,[45] the ubiquity of EHR data makes it vital that we figure out how to leverage this resource for purposes of identifying early ADRD.

Relatively few research studies have explored the use of both the EHR data and ML algorithms to detect ADRD.[21-24] Two studies developed electronic search algorithms for identifying ADRD from EHR notes.[22, 23] Another study examined the impact of combining natural language processing with the presence of ADRD-related diagnosis codes and ADRD medications.[21] A fourth study used Bayesian network to develop an algorithm that reached 80% accuracy related to the diagnosis of ADRD based on five cognitive exams that are not available at scale in most EHR systems used in primary care practices.[24] While the accuracy from using Bayesian networks is good, a significant amount of neuropsychological data, computation, feature engineering and expert-guided bootstrapping is required in order to use this type of ML

algorithm. The use of ML algorithms that require capturing detailed neuropsychological or invasive biological data from large numbers of older adults in primary care practice is simply not scalable nor sustainable. While the few studies that used ML algorithms and existing data in the EHR demonstrate utility in identifying patients diagnosed with more advanced ADRD, they do not support the early screening of patients at risk for developing ADRD. Thus, balancing the capability of the most accurate ML algorithms, the available data within the current EHR systems and the focus on ADRD screening is the essential core of developing and implementing a low-cost scalable early detection approach for ADRD in primary care practices. At Indiana University, Drs. Boustani and Ben-Miled leveraged the EHR data and the advantages of ML algorithms to develop a Passive Digital Marker (PDM) for early detection of ADRD with 80% accuracy for one-year and three-year prediction horizons.[10]

A.4. Do patient reported outcomes (PRO) improve early detection of ADRD? Previous approaches to early ADRD detection have included the use of cognitive screening tests and the use of invasive or expensive biological markers.[2,3,5, 13, 25,26] However, up to 38% of patients refuse cognitive screening tests,[17, 27-32] these tests fail to detect ADRD in individuals with high cognitive capacity, and their cultural, language and educational biases lower their accuracy in underrepresented groups.[26] Using biomarkers for early detection of ADRD are not scalable due to their invasive nature (lumbar puncture); their cost (MRI, PET); and their accessibility (rural or underserved areas). Furthermore, ADRD can be insidious in its onset with symptoms fluctuating over time.[3] Thus, Patient Reported Outcome (PRO) approaches can overcome the above barriers for early detection of ADRD in primary care practices. PROs can monitor ongoing symptoms of ADRD as well as how these symptoms affect patient functioning. They can create efficient and cost-effective clinical encounters with providers while also empowering patients and family caregivers to engage in early detection of ADRD.[26, 33, 34] Dr. Galvin's (a Co-PI on this proposal) team developed and tested the Quick Dementia Rating Scale (QDRS) as a PRO tool for both early detection and staging of ADRD with 85% accuracy for ADRD diagnosis.[11] The QDRS is a 10-item, multiple choice questionnaire that takes 2-3 minutes to complete and is highly correlated with Gold Standard measurements including the Clinical Dementia Rating scale, neuropsychological testing, and ADRD biomarkers (MRI, CSF, PET).[11,55] Completion of the QDRS can offer several advantages above and beyond what is captured through EHR review including (a) capture of non-memory symptoms (e.g., orientation, problem-solving, daily functioning) that are both disturbing to patients and families and are more likely to be accepted as a change that requires medical attention; (b) provide information about the patient's real-world functioning; (c) provide information at visits for new patients where prior EHR data may not be available; (d) capture of progression over time; and (e) allow for staging of ADRD in a brief, valid, and time- and cost-effective manner. [11,26, 55]

B. INNOVATION:

The proposal is innovative for the following reasons.

B.1. The proposal combines the multifunctionality of a PDM relying strictly on near-universal routinely-collected EHR data with the QDRS, a tool that depends on very feasible, scalable and low-cost patient reported data. Such a combined approach benefits from directly identifying patterns of health from the EHR indicative of early ADRD, while also supplementing known limitations of EHR data through direct capture of PROs from patients (i.e., in the absence of efforts by licensed professionals). These complementary combined approaches replicate ‘real-world’ approaches common to primary care (review of records plus patient interview). Thus, the proposal directly addresses the objective of the NIA call for “the development of low cost, effective, and scalable approaches for early detection of ADRD” (RFA-AG-20-051).

B.2. The PDM leverages two types of EHR-data; the structured EHR data including demographic information, diagnoses, and prescriptions; and the unstructured data from free text documents such as patients’ visit notes, progress notes, and medication notes. The PDM applies a novel differential clustering technique to the unstructured notes in order to automatically derive a lexicon to translate the unstructured data into a feature vector following the bag-of-word approach. The PDM then used features from the structured and unstructured EHR routine care data to train a Random Forest ML algorithm.[10] The Random Forest is an ensemble learning technique that can handle high dimensional and noisy feature spaces.[35] This characteristic is especially important when using EHR data. In fact, most previous predictive ADRD models have been derived by using structured data from targeted medical tests such as MRI and cognitive tests.[36-44] These tests are costly (in term of money or time) to administer and as a result, most of the cohorts used to develop the associated ML algorithms were limited in number. [36-44] By selecting the appropriate ML algorithm and developing novel and scalable differential clustering techniques, the PDM was able to leverage the vast amount of EHR data to develop an algorithm with a prediction horizon of 1 to 3 years.[10] The PDM compared favorably to the results of a recent study of the Health and Retirement Study where regression-based diagnostic algorithms combined with patient reported data produced areas under the curve (AUC) of 0.84 to 0.85 in validation samples.[44] Thus, the performance of the proposed PDM is commendable, especially given that it represents a *screening* algorithm with a 1 to 3 years prediction horizon, as opposed to a diagnostic one.

B.3. The application proposes to conduct three complementary studies in very diverse rural, suburban, and urban primary care practices with high percentages of African Americans (Indiana sites) and Hispanic individuals (South Florida) with low socioeconomic status. Thus, the PDM and the QDRS will be deployed in two sites in order to validate their generalizability for diverse and underserved patient populations.

C. PRELIMINARY WORK

C.1. Our interdisciplinary scientific team (see key personnel): Under the leadership of Drs. Boustani, Galvin, and Ben-Miled, we have assembled an impressive cadre of senior and junior scientists with expertise in ADRD screening and diagnosis (Drs. Boustani, Galvin, and Fowler); Machine Learning (Dr. Ben-Miled and Kleiman); Psychometrics (Drs. Monahan, Boustani, and Galvin); Medical informatics (Drs. Dexter, Boustani, and Grout); and Implementation Science (Boustani, Fowler, and Grout). The members of this team have a track record of collaborating in conducting research activities similar to the proposed activities within this application including creation and validation of PROs and utilization of ML algorithms to improve ADRD detection through the EHR.[10, 17,19, 27-29, 31, 33] In the following section, we summarize the results of preliminary and relevant studies conducted by members of this team which informed the design of our proposed studies. Such preliminary studies are selected from a large repository of research conducted by this team (see scientific contributions within the key personnel).

C.2. The limited impact of the current AWV on early detection of ADRD: We conducted a retrospective matched cohort study to examine the impact of the AWV on the detection of ADRD.[19] The cohort included 5% sample of Medicare beneficiaries continuously enrolled for 12 months before and after an index ambulatory visit with no evidence of ADRD before the index date. The matching process resulted in 66,399 matched pairs of beneficiaries with and without an AWV. Outcomes included 12 months post-index visit claims-based measurements of new ADRD diagnoses, medications for ADRD, and cognitive care-related diagnostic work up such as neuropsychological testing, brain imaging, blood tests for thyroid-stimulating hormone (*TSH*), serum B12, folate, or syphilis. *There were no clinically relevant differences between the AWV and control groups in the rates of incident ADRD diagnoses (6.16% versus 6.86%, p<0.001) and the initiation of ADRD medications (1.00% versus 1.08%, p=0.148).* While the AWV was correlated with an increase in some measures of cognitive care (e.g., TSH, B12, and folate testing), we did not find that the current AWV increased recognition of undetected ADRD. Enhancing the AWV with data from the PDM, the QDRS, or both may overcome current AWV shortcomings.

C.3. We can enroll a large number of older adults attending diverse rural, suburban, and urban primary care practices: We conducted a single-blinded, 2-arm, randomized controlled trial in urban, suburban, and rural primary care practices.[17] We approached approximately 7,000 eligible older adults to enroll and randomize 4,005 subjects (enrollment rate of ~60%) to ADRD screening (n=2,008) or control (n=1,997). Patients were screened using the Memory Impairment Screen[46,47] or the Mini-Cog[48] and referred for a voluntary follow-up diagnostic assessment if they screened positive on either or both screening tests. Primary outcomes were health-related quality of life measured with the Health Utilities Index[49] at 12 months, depressive symptoms measured with the Patient Health Questionnaire-9[50], and anxiety symptoms measured with the Generalized Anxiety Disorder 7-item scale[51] at 1 month. The mean age was 74.2 years (standard deviation 6.9); 2,257 (66%) were female and 2,301 (33%) were minority population. The trial did not detect any harm of dementia screening.

The current proposal directly leverages lessons learned from this prior study. Our previous trial did not adequately leverage the trusted relationship between the patient and her primary care team. Screening results were not integrated within the primary care clinic flow. The disclosure of the results was conducted by the research team rather than the primary care team, and the research team was responsible to coordinate the referral for the diagnostic assessment not the primary care physician. Indeed, the current proposed PDM and QDRS will be fully integrated within the AWV and the clinical flow of the primary care practices.

C.4. Using ML algorithm and routine EHR data to develop a Passive Digital Marker (PDM) for early detection of ADRD: The current approaches of using cognitive tests and biomarkers for early detection of ADRD are not scalable due to the high patient refusal rates (cognitive and biomarker tests), their invasive nature (lumbar puncture); their cost (MRI, PET); and their lack of accessibility in rural or underserved areas.[13, 17, 25-32] In order to address this gap, we developed a ML algorithm that can predict ADRD one year and three years prior to its onset by using routine care EHR data.[10] The algorithm was trained using structured and unstructured data from three EHR datasets: diagnosis (Dx), prescriptions (Rx), and medical notes (Nx). Individual algorithms derived from each of the three datasets were developed and compared to a combined one that included all three datasets.

C.4.a. Data Preprocessing: The ML algorithms were trained and tested by using EHR data of incident ADRD cases and non-ADRD controls. Cases of prevalent Mild Cognitive Impairment (MCI) or ADRD were excluded in order to avoid any potential for positive predictive bias. The diagnosis date (index date) for each case was established and 3 to 4 matching controls were identified. The matching criteria were based on birth year, gender, race and index date (within 6 months). Only patients who had at least one encounter per year were retained for a total of 2,159 cases and 11,558 controls from 15 and 25 different institutions in Indiana, respectively. With respect to race and gender, the distribution of the cases and controls (Table 1) was similar, thus limiting any gender or race bias among the two classes. However, within a class, there are more females and more patients of white race than the other gender and races.

Table 1: Demographics of the dementia cases and healthy controls.

	African American		White		Other	
	Male	Female	Male	Female	Male	Female
Cases	10%	21%	23%	41%	2%	4%
Controls	11%	19%	24%	42%	1%	3%

C.4.b. Feature Engineering: The features (variables) of the algorithm include demographic features (i.e., age, gender, race) and medical features extracted from the prescription (Rx), diagnosis (Dx) and medical notes (Nx) categories of the health record. Feature engineering followed a different approach for each dataset. Each Rx feature corresponds to a drug group according to the Generic Product Identifier (GPI) classifier for a total of 100 features, and the value of the feature is the number of times a medication from the given drug group was prescribed to the patient within the algorithm's period. Medical disorders identified by experts (Table 2) defined the 19 features of the Dx model where the feature values are the count of the diseases in each disease group during the algorithm's period. The Nx features are obtained from medical notes. These notes are unstructured and include different administrative and medical report types. After the exclusion of administrative reports, the remaining 340 medical report types were used to construct the Nx dataset. A new clustering algorithm that leverages the differential frequency between cases and controls was developed. This process resulted into 110 bag-of-words clusters where each cluster corresponds to a feature. The processing of the raw data took into consideration missing values and erroneous data especially in the case of the NX, where errors are more common due to human-entry of data. As such, the algorithm does not rely on extensive pre-processing of the raw data.

Table 2: List of dementia related disorders or disease groups in Dx.

Angina	Chronic Ischemic Heart Disease
Anxiety	Transient Ischemic Attack
Abnormal Weight Loss	Transient Ischemic Attack Related Syndromes
Bipolar Disorder	Other Acute Ischemic Heart Disease
Depression	Stroke/Cerebral Infarction
Insomnia	Acute/Subsequent MI
Hypercholesterolemia	Hemorrhagic Cerebrovascular accident
Hypertensive Disorders	other Cardiovascular diseases
Schizophrenia	Claudication/Atherosclerosis
[illegible]	

C.4.c. Development and Evaluation: Random Forest was used to develop two algorithms (or models) for each dataset: The 1Yr model is trained and tested by using the available health records during the period (index date - 10 years) to (index date - 1 year). Similarly, the 3Yr model is trained and tested by using the patient records during the period (index date - 10 years) to (index date - 3 years). The mean and standard deviation of the accuracy, sensitivity, and specificity across the 5 groups in a 5-fold cross validation of the models is shown in Table 3. The last row of this table corresponds to a combined model that was developed by using all three data sets (i.e., Nx, Rx, Dx). The 1Yr models have higher accuracy, sensitivity and specificity than the corresponding 3Yr models. This is expected since the former models have access to more recent records. Among the three individual models, the Nx model has the highest accuracy, sensitivity and specificity. Moreover, the accuracy of the combined model (RDNx) is higher than any of the individual models. This is an indication that the Nx features have a higher ADRD prediction entropy compared to the Rx and Dx datasets; nonetheless, the Rx and Dx features make a significant contribution to the combined model.

Table 3: Accuracy, sensitivity and specificity for the 1Yr and 3Yr dementia prediction models trained by using different data sets. For each metric, the entry in the table corresponds to the mean value of all groups in a 5-fold cross validation and the number in parenthesis is the standard deviation across the groups.

	Model	Accuracy (%)	Sensitivity (%)	Specificity (%)
Rx	*1Yr*	70.39 (0.88)	68.94 (2.35)	70.46 (0.98)
	3Yr	65.63 (1.24)	65.00 (3.91)	65.65 (1.43)
Dx	*1Yr*	65.21 (0.74)	66.06 (2.44)	65.18 (0.80)
	3Yr	62.91 (1.71)	63.80 (4.26)	62.87 (1.95)
Nx	*1Yr*	74.07 (0.98)	72.01 (1.72)	74.16 (1.01)
	3Yr	70.13 (2.65)	67.31 (3.28)	70.25 (2.85)
RDNx	*1Yr*	77.43 (1.89)	76.01 (1.88)	77.49 (2.02)
	3Yr	73.50 (2.03)	70.93 (2.18)	73.61 (2.17)

The choice of Random Forest for the ML technique was highly dependent on the characteristics of the data source. Compared to other techniques, Random Forest can accommodate a large number of features and these features can be either categorical or numerical. The resulting model is interpretable offering the ability to use the decision path within the model to support the specific outcome for each individual patient. This is essential for adoption by clinicians and patients.

Other ML techniques were considered. Bayesian Network models are highly interpretable. However, they require significant engineering effort and are computationally demanding especially when the number of features is large, making these models impractical for use with EHR data.[24, 35, 45] Our own preliminary study compared Random Forest to Support Vector Machine and Artificial Neural Network. Support Vector Machine had a comparable accuracy, but it is not as interpretable as Random Forest.[35, 45] Artificial Neural Network had a lower accuracy primarily because the number of available patient records was not sufficient to adequately train a high dimensional deep neural network. Furthermore, the Artificial Neural Network model is not interpretable.[24,35, 45] We also developed a model using linear regression for the prediction of ADRD.[52] The linear regression model was limited by the number of predictors it can accommodate compared to the Random Forest (i.e. 232 predictors) and it had a lower sensitivity.[10,52]

C.5. The development and validation of the Quick Dementia Rating Scale (QDRS) as a patient reported outcome (PRO) tool for ADRD screening and Staging. Early ADRD detection in a healthcare delivery system including primary care is challenging because many rating scales take significant time and require specialized training.[3,5,13,25,26] To address this, we developed several culturally- and linguistically-sensitive patient reported outcome (PRO) tools as scalable and sustainable approaches for the early detection and staging of ADRD.[11, 33, 53-55] The first of these scales was *the AD8* as a brief screen to detect ADRD in the community that now has global applications.[53,53] Although sensitive to cognitive impairment, the AD8 lacked properties for staging and did not permit differential diagnosis.[53,54] As the AD8 was designed as a cross-sectional screening instrument, for longitudinal study, we developed and validated the *Quick Dementia Rating Scale (QDRS).*[11,55] The QDRS is a 10-item, multiple choice questionnaire that takes 2-3 minutes to complete. It offers a range of scores from 0-30; scores 0-1.5 signify normal cognition, scores

2-5.5 signify mild cognitive impairment, scores 6-12.5 signify mild ADRD, scores 13-20 signify moderate ADRD, and scores 20.5-30 signify severe ADRD. It provides a brief, valid, and reliable assessment regarding the presence and severity of a cognitive problem and demonstrates differential scoring across different ADRD etiologies (Table 4).[11,55]

The QDRS was tested and validated against Clinical Dementia Rating (CDR), cognitive testing, and Gold Standard measures of cognition, function, and behavior. QDRS scores increased with higher CDR staging and poorer neuropsychological performance (p's <.001). The QDRS demonstrated excellent known-groups validity (p's<.001); construct validity against Gold Standard (p's<0.004); and reliability (Cronbach α: 0.86-0.93). The QDRS is now being used by other investigators in prevention studies[55] and in clinical trials (i.e., Biogen) for subject screening with translations being validated in Spanish, Korean, Chinese, and Portuguese.

Table 4: Properties of QDRS by Cognitive Status and Dementia Etiology (adapted from Galvin JE, Alz Dem; DADM 2015)

	Controls	MCI	AD	LBD	VCID	FTD	p-value
Age, y	70.1 (7.6)	76.2 (8.9)	79.8 (7.5)	78.4 (7.7)	77.2 (6.2)	72.7 (8.2)	.001
Education, y	16.7 (2.4)	15.9 (3.0)	15.2 (2.9)	14.5 (3.6)	14.8 (3.4)	16.8 (3.3)	.28
Charlson Comorbidity Index	1.7 (1.9)	1.9 (1.3)	2.3 (1.3)	2.4 (1.5)	2.6 (1.1)	2.0 (1.3)	.13
Clinical Dementia Rating (CDR)	0.2 (0.3)	1.9 (1.6)	1.0 (0.6)	1.5 (0.9)	1.7 (0.9)	0.8 (0.8)	<.001
CDR-Sum of Boxes	0.03 (0.1)	0.4 (0.3)	5.7 (3.3)	8.8 (5.2)	9.3 (6.3)	5.2 (4.7)	<.001
Mini Mental State Exam	28.7 (1.6)	26.1 (3.3)	19.6 (5.5)	18.2 (7.7)	19.7 (6.0)	23.6 (1.4)	.005
Functional Activities Questionnaire	0.0 (0.0)	3.6 (4.2)	10.5 (8.5)	17.1 (10.1)	16.6 (13.9)	8.1 (9.9)	.001
QDRS Total	0.3 (0.5)	3.5 (2.7)	7.2 (5.1)	11.7 (6.9)	11.6 (7.8)	7.4 (6.3)	<.001
QDRS Cognitive Subscale	0.2 (0.3)	1.5 (0.9)	3.1 (1.9)	4.5. (2.6)	2.8 (2.3)	2.7 (2.4)	.005
QDRS Behavior Subscale	0.2 (0.3)	2.0 (2.0)	4.2 (3.5)	7.5 (4.9)	8.8 (5.9)	5.4 (4.8)	<.001

Although initially tested as an informant rating, we examined the utility of the QDRS as a PRO tool. We compared informant QDRS, patient QDRS, and Gold Standard CDR and CDR-Sum of Box in 254 consecutive dyads presenting to neurology clinics. The QDRS ratings were not considered in the Gold Standard evaluation and were completed prior to the office visit by the informant and patients. Cronbach alpha was examined as a measure of internal consistency. The internal consistency of the two versions of the QDRS were: Informant QDRS: 0.939; Patient QDRS: 0.922, supporting that both versions have excellent psychometric properties. Intraclass correlation coefficients (ICC) were used to assess interscale reliability comparing CDR domains, Sum of Box, and global scores with the QDRS correcting for chance agreement. The ICC examines the proportion of responses in agreement in relation to the agreement expected by chance. An ICC between 0.55 and 0.75 is considered good agreement, whereas an ICC greater than 0.76 is considered excellent.

Table 5: Construct reliability between QDRS versions and CDR			
	Inf QDRS – Pt QDRS	Inf QDRS – CDR	Pt QDRS - CDR
Memory	.756	.754	.499
Orientation	.783	.787	.699
Decision making	.750	.728	.742
Activities outside home	.803	.862	.780
Activities inside home	.822	.861	.759
Personal hygiene	.918	.887	.855
Behavior	.716	-----	----
Language	.839	----	----
Mood	.729	----	----
Attention	.749	----	----
Total QDRS	.887	----	----
QDRS-derived CDR-SB	.889	.909	.831
QDRS-derived CDR	.770	.826	.740

The informant and patient versions of the QDRS have high degrees of agreement, and both versions show good to excellent agreement with Gold Standard CDR (Table 5).

We then looked at concurrent (criterion validity) comparing the mean performance on each Gold Standard measure of cognition (e.g., CDR, CDR-Sum of Box, neuropsychological testing), function (i.e., FAQ), behavior (e.g., NPI, Hospital Anxiety and Depression Scale), and caregiver characteristics (e.g., burden, depression) with the QDRS. Both informant and patient versions of the QDRS demonstrated strong relationships with clinical, cognitive and psychological variables. Strength of association was comparable to that seen for CDR and CDR-Sum of Box. Further, we compared the QDRS to ADRD biomarkers (Galvin et al, manuscript under review 2020). Total QDRS scores as well as individual domain scores (e.g., memory, orientation, decision making, daily functioning) were significantly correlated with CSF Ab42, Ab42/tau ratios, PET PiB index, MRI total gray matter and hippocampal volumes (all $p<.01$ correcting for multiple comparisons). The addition of the QDRS as a PRO to the PDM is an enhanced complementary approach to capturing real-world patient functioning, cognition, mood and behavior not routinely captured in the EHR, and provides important information regarding patients at the initial visit where EHR data may not be available.

D. RESEARCH APPROACH

D.1. General Overview and Design (see figure): Our research design is predicated on the notion that patient screening would appropriately identify a more targeted group for referral for appropriate diagnostic services. We will conduct three complementary studies to evaluate the effectiveness of the PDM, the QDRS or the combined (PDM + QDRS) approaches for early detection of ADRD. These approaches will be embedded within the EHR systems of diverse rural, suburban, and urban primary care practices in central Indiana and south Florida. The first study will be a clinical validation study (stage III) of the three approaches; the PDM, the QDRS, and the combination of both (the PDM + QDRS). The approaches will be clinically validated in real-world and diverse clinical settings in comparison to the gold standard of ADRD diagnosis used in the NIA-funded ADRD Research Centers across the nation. The gold-standard evaluations will allow us to determine the accuracy of the screening approaches and compare their performances. The other studies will be two independent pragmatic cluster-randomized controlled comparative effectiveness trials (phase IV) of the approaches embedded within the AWV (PDM within AWV, PDM + QDRS within the AWV) in comparison to the current AWV only process (AWV without PDM or QDRS). The two trials will be conducted in two separate healthcare systems; the first trial will be conducted at Eskenazi Health and within 12 months a second replicated trial will be conducted at University of Miami Health. Both trials will have identical methodology and would compare the performance of the three approaches in increasing the incidence rate of new ADRD subsequently documented in the EHR by the primary care practices.

D.2. Clinical Setting for both the Clinical Validation Study and the Pragmatic Cluster-Randomized Controlled Comparative Effectiveness Trials: All studies will be conducted in diverse rural, suburban, and urban primary care practices in central Indiana and south Florida. Such primary care practices include 10 federally qualified health centers affiliated with Eskenazi Health in Indianapolis and 10 primary care practices in South Florida affiliated with University of Miami (UHealth). Eskenazi Health is one of the largest safety net integrated healthcare systems in the nation serving underprivileged older residents of Marion County including African Americans (50%) and dual eligible Medicaid and Medicare beneficiaries with a low socioeconomic status (50%). The UHealth has primary care practices serving older adults residing in Miami-Dade, Broward, and Palm Beach Counties and serving underrepresented groups of African Americans (19%) and Hispanic (45%) populations throughout South Florida. Our interdisciplinary research team has long-standing relationships with many sites in the two states. In late 2019, we published the results of a randomized controlled trial evaluating the

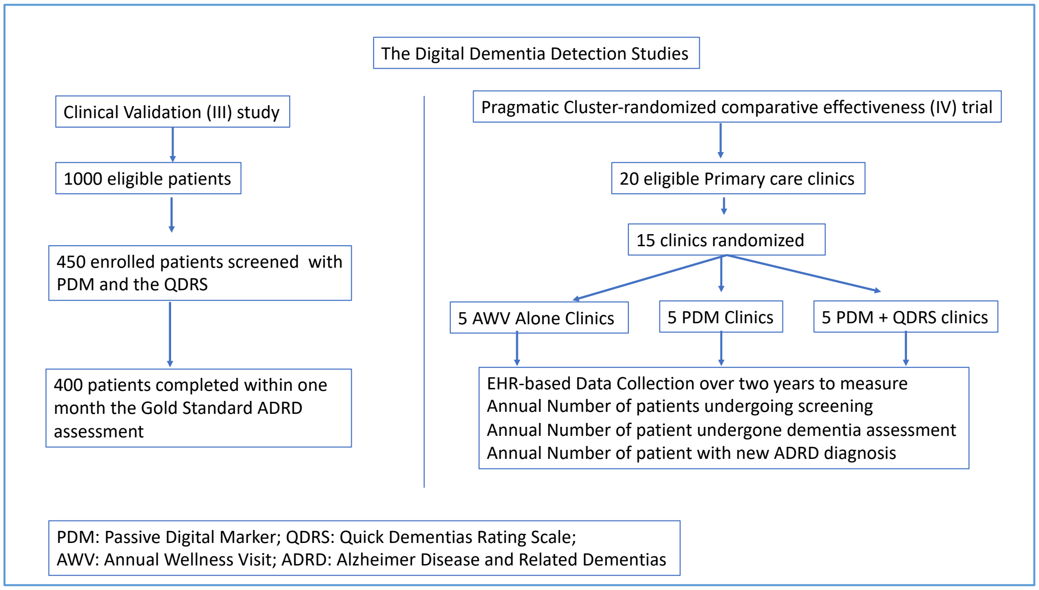

benefits and harms of ADRD screening among 4,000 older adults attending rural, suburban, and urban primary care practices.[17]

D.3. Subjects Recruitment into the Clinical Validation (Stage III) Study: To recruit patients from the above clinical setting into our clinical validation study and using similar processes to our previous studies,[12,15,17, 27-33] trained research assistants will work with the practice manager from each primary care practice to identify potential subjects who meet the inclusion and exclusion criteria outlined in the table below. The research assistants will make initial contact via a phone or face-to-face at the primary care practices to garner interest in participating in the study and verify if the patient meets the inclusion criteria (see Table 6). Based on the literature, our own pilot data, and our power analyses (described below), we anticipate approaching 1,000 eligible subjects to enroll and complete data on 400 subjects. Rolling enrollment will take place over 18 months with an average monthly enrollment of 23 subjects per month.

Table 6. Inclusion and exclusion criteria

**Inclusion criteria**	**Exclusion criteria**
65 years or older	Prior ADRD or mild cognitive impairment diagnosis as determined by ICD-10 code
At least one visit to primary care practice within the past year	Evidence of any history of prescription for a cholinesterase inhibitors or memantine.
Ability to provide informed consent	Has serious mental illness such as bipolar or schizophrenia as determined by ICD-10 code
Ability to communicate in English or Spanish	Permanent resident of a nursing facility
Available EHR data from at least the past three years	

D.4. Primary Care Practices Recruitment into the Pragmatic (Phase IV) Trials: We will approach the practice manager and the physician leader of each primary care practice to randomize their site into one of three early detection approaches; AWV without PDM or QDRS; AWV with PDM; and AWV with both PDM and QDRS. We have received approval from the leadership of both Eskenazi Health and UHealth. The PDM and the QDRS will be embedded within the AWV process for each practice. The current rate of using the AWV across the country is increasing from 14% in 2012 to more than 60% in our own practices. We will obtain a waiver of informed consent from the local Institutional Review Board to review retrospectively (after at least one year of screening) the EHR systems of each practice to calculate the annual rate of new ADRD diagnosis and other EHR data to measure processes of diagnostic assessment following the AWV including screening rate, referral rate for diagnostic assessments for early ADRD and patient acceptance rate of undergoing such assessments.

D.5. Interventions and Control for the Pragmatic (Phase IV) Trials: Our interdisciplinary scientific teams developed and tested both the PDM and the QDRS. Drs. Boustani and Ben-Miled leveraged the widely available EHR data and the advantage of ML to develop the PDM with an approximate 80% accuracy for one-year and three-year prediction horizons.[10] Dr. Galvin's team developed and tested the QDRS as a practical 2-3 minute patient reported outcome (PRO) tool for both early detection and staging of ADRD.[11]
Each pragmatic trial will randomize primary care practices into one of three approaches for early detection of ADRD at (1:1:1) ratio. These approaches include the current state of using AWV in primary care (the control group), the incorporation of the PDM into the AWV, and the combination of PDM and QDRS (Passive + QDRS). All of the three approaches will be

implemented within the current Medicare funded AWV and will be integrated within the provider clinical encounter to accommodate the clinical flow and data capture and results display needs of the primary care practices. To randomly assign each practice to one of the three screening approaches, we will use a computer-generated randomization scheme.

D.6. Integrating the PDM and QDRS within the Epic EHR: We will leverage the processes and technologies currently and successfully in use every day in our clinical partner's primary care practices. These practices at both sites use Epic as their EHR and its related computerized decision support (CDS) engine and its patient-completed survey platforms. As indicated in the preliminary studies, EHRs contain detailed clinical data that can be accessed to run the PDM algorithm. EHRs also have a platform for patient reported outcome (PRO) survey in which we will integrate the QDRS for the selected primary care practices. The EHR-based CDS system will display the results of the screening to clinicians to promote the interpretation of the results by healthcare providers and patients. The Regenstrief Institute (see Resources section) has decades of experience in successful CDS research and implementation. Over the past three years, we have partnered with our Epic EHR clinical informatics colleagues to deploy various CDS tools such as patient reported surveys, predictive algorithms for social frailty, and deprescribing recommendations.[56-67] These CDS tools provide clinicians with a graphical summary of the results with a focus on the factors driving these results. A similar approach will be adopted in this study, tailored through the extensive configuration options available in the Epic CDS engine. Patient registration at the primary care clinic would trigger the CDS process including patient completion of the QDRS. The CDS engine will also query the data sources for the required input features specific to the patient and invoke the PDM ML algorithm. Results will be displayed within the EHR user interface with an emphasis on the key features of the model that explain the final outcome for each individual patient, including actionable clinician-directed recommendations related to appropriate diagnostic work-up. The proposed workflow will be deployed in the two healthcare systems (Eskenazi Health and UHealth) and integrated within the flow of the primary care practices. We have received the approval from leadership at both sites for the deployment of both the PDM and the QDRS, as described in their letters of support. The technical infrastructure (including servers, EHR and CDS configuration options, and data connections) as well as the security and governance agreements already exist and therefore pose no threats to project objectives or risk of delayed timelines.

D.7. Data Sources and Collection: We will collect EHR data from all patients enrolled in the clinical validation study (identified data after obtaining informed consent from subjects) and from patients receiving care in all of the practices enrolled in the two pragmatic trials (de-identified data after obtaining consent from the practice leadership and receiving waiver from the IRB). For the clinical validation study, the research team will conduct a gold-standard ADRD assessment on all patients screened by the PDM and the QDRS.

D.7.a. ADRD Diagnostic Assessment: The ADRD assessment is modeled on the Uniform Data Set (UDS) 3.0 from the NIA Alzheimer's Disease Center program.[68,69] The assessment will be performed face-to-face or via Zoom at research centers in Indianapolis and Miami. To accommodate subject preference, the assessment may be conducted at the subject's home for convenience. We have used such assessments for more than a decade with excellent quality assurance and completion.[11,12, 33,53-55, 61] The ADRD assessment will include the following data that will be entered into a REDCap database, a browser-based, metadata-driven software and workflow methodology for designing clinical and translational research databases.

<u>Subject interview and Medical Record Review</u> will be used to collect information on demographics, primary language, alcohol/tobacco/substance use history, and family history. We will review the

patient's medical records for the presence of comorbid conditions, medications, vitamin B12 level, thyroid test, syphilis tests, and brain imaging. When relevant to making a diagnosis and unavailable in the patient's record, laboratory tests or brain imaging will be requested in collaboration with the primary care clinicians.

Medical Evaluation: A detailed physical and neurological examination will be completed by trained research nurses. The Modified Hachinski Scale will assess vascular risks.[68,69]

Cognitive Status: The Montreal Cognitive Assessment will be used for a global screen.[70] The rest of the cognitive battery includes the Uniform Data Set (UDS) battery used in the NIA Alzheimer Disease Centers: 15-item Multilingual Naming Test (naming);[69] Animal naming and Letter fluency (verbal fluency);[69] Craft Story for paragraph recall testing of episodic memory[69] supplemented with the Hopkins Verbal Learning Task (episodic memory for word lists – immediate, delayed and cued recall);[71] Number forward and backward and Months backwards tests (working memory);[69] and Trail making A and B (processing and visuospatial abilities).[72] The Geriatric Depression Scale is performed for assessment of mood.

Informant-Based Rating Scales: The CDR[73] will be used for global staging, the Neuropsychiatric Inventory (NPI) to assess behavior,[74] and FAQ[75] to assess activities of daily living.

Determination of Cognitive Status: Global rating scales will be combined with cognitive performance and medical record review to assign individuals to the following diagnostic categories after a consensus conference: No Cognitive Impairment (NCI), Subjective Cognitive Impairment (SCI), Mild Cognitive Impairment (MCI) or ADRD. MCI categories will be attributed based on the number of cognitive domains affected (single vs multiple; amnestic vs non-amnestic). ADRD cases will be assigned etiologies (AD, LBD, VaD, FTD, or mixed).

Professional research assistants will undergo a detailed training by Drs. Galvin and Boustani and will be certified in conducting the UDS assessment. Similar to our previous process from various research studies, [12,17,76] we will use several incentives to encourage patients who screened positive to undergo the diagnostic assessment. First, we will work with the patient's physician to recommend the assessment. Second, the study personnel will provide information on the importance of the assessment. Third, the assessment will be offered free of charge. If transportation is a barrier, the patient will be offered free transportation or home-based assessment. Fourth, patients and their care partners will be reimbursed for their time with a $50 gift for each.

D.7.b. Incidence of ADRD for the pragmatic cluster-randomized comparative effectiveness clinical trials: For the two pragmatic trials and using similar approach from previous studies,[12, 19] we will use data captured by the EHR to assess for two outcomes. The primary outcome measure will be any new ADRD case identified (documented in the EHR) within 12 months of the Annual Wellness Visit (index visit). The secondary outcome measures will be any services related to cognitive diagnostic assessment in the post Annual Wellness Visit (index) period that providers may order to diagnose or exclude ADRD. Specifically, the metrics of diagnostic assessment will be evaluated as proportions of patients with a record of 1 or more of:

- Laboratory tests for TSH, serum B12, folate, or syphilis; individually or combined at any point during the 90 days after index
- Neuropsychological testing, including testing by psychologist or physician, technician administrator, computer, or other providers during the 12 months after index date
- Brain imaging testing (computed tomography, magnetic resonance imaging, positron emission tomography, magnetic resonance angiogram) of the head and neck, brain, or skull during the 12 months after index date
- Medications approved for management of ADRD (cholinesterase inhibitors, memantine) during the 12 months after index date

D.8. Analysis Plan

D.8.a. Primary Aim 1: *Evaluate the predictive performance of the PDM, the QDRS, and the combined approach in the early detection of ADRD, compared to the gold standard diagnoses*.

For Primary Hypothesis 1, the screening approaches will be validated by evaluating the Area Under the Curve (AUC) for the PDM, the QDRS, and the combined approach. For the purpose of Aim 1, the PDM and the QDRS will be administered in the same sample of patients to increase power while retaining internal validity of conclusions.[77] The AUC will be calculated from the plot of the sensitivity (y-axis) by 1 – specificity (x-axis) across all possible discrete screening score values. For the combined approach, screening will be considered positive if either PDM or QDRS are positive. The gold standard diagnosis will be determined as described in **D.7.a.** We hypothesize that the AUC, as well as the overall % accuracy of classifications, will be 0.85 or greater for the combined method, and 0.75 or greater for PDM and the QDRS.

For Secondary Hypothesis 2, dependent AUCs will be compared using the method[78] that extends DeLong et al.'s nonparametric method[79] to be valid for clustered data by using the Rao and Scott concepts for clustered binary data.[80] The AUCs of different methods are dependent in the Aim 1 study because they are calculated on the same sample of patients. We hypothesize that the combined approach of PDM and QDRS will have statistically better AUC than PDM or the QDRS alone. In addition, the screening approaches will be compared on the overall % accuracy of classification (i.e., a simple proportion based on 0/1 data for each person) using nonlinear mixed models to account for correlated outcomes due to dependent methods and practice-clustered data.

Potential problems & alternative strategies: The distributional assumptions for the binary outcomes are minimal, which will decrease the need to consider alternative analytic methods and models. However, to ensure the analytic methods appropriately account for the clustering effect when estimating standard errors and p-values, we will perform bootstrap estimation (i.e., using 1,000 datasets resampled with replacement at the person level) of the standard errors and confidence intervals surrounding the estimated values for AUC and % accuracy, and the estimated values for the difference in these values between screening approaches. In addition, if missing data occur, we will impute data using the simulation-based "Multiple Imputation" method,[81] in which multiple complete datasets with plausible values for missing data are generated using model-based prediction, and analytic results from imputed datasets are aggregated to calculate appropriate standard errors and p-values in the presence of imputed data. We will use 100 imputed datasets, even though 10 to 50 are considered sufficient.[81] Inclusion of potential auxiliary variables in the imputation model will be considered. A sensitivity analysis will be performed using participants with complete data.

D.8.b. Primary Aim 2: Evaluate the practical utility and effect of the PDM, and the combined methods in improving the annual rate of new documented ADRD diagnosis in primary care practices.

For Primary Hypothesis 1, three pragmatic screening approaches (AWV alone, AWV + PDM, and AWV + PDM + QDRS) will be compared on the annual incidence rate of EHR documented ADRD using nonlinear mixed models to account for clinic-clustered data. In the two pragmatic trials, different patients are randomized (via their clinics) to each of the 3 arms, making the outcomes independent between the three screening approaches; however, data will still be correlated for Aim 2 due to clustering of patients within clinics, which will be handled by random effects in the mixed models. We hypothesize the incidence rate will be 6% using AWV alone[19]

and 13% with the screening approach that combines AWV + PDM + QDRS. Incidence rates for all arms, including for AWV + PDM, will be calculated along with their 95% confidence interval.

For Secondary Hypothesis 2, the analyses will be the same as described for Hypothesis 1 of Aim 2, except that the outcome will be acceptance rates for recommended ADRD diagnostic work-up following positive screen. We hypothesize the acceptance rate will be 44% using AWV alone[17] and 66% with the screening approach that combines AWV + PDM + QDRS.

Potential problems & alternative strategies: Same as those described for Aim 1 above.

D.8.c. Sample Size & Power: Calculations were performed with PASS, a comprehensive power and sample size software.[82] All calculations assume two-sided tests and alpha = 0.05. For study 1 (i.e., Aim 1), a sample size of 15 cluster pairs consisting of 400 persons (26.667 persons per cluster) will provide 81% power to compare population values of 75% versus 85% in the outcome of % accuracy of classification. This is based on using nonlinear mixed models to compare two paired (i.e., dependent) proportions. The proportions are considered dependent instead of independent because all three screening approaches for study 1 will be performed in the same sample of 400. This approach was selected because it will increase power by allowing participants to serve as their own controls.[77] Moreover, it is appropriate for a diagnostic validity study to compare methods in the same sample (i.e., method contamination is not an issue).[77] This calculation assumes that participants are recruited from 15 clinics (i.e., clusters) and that the within-pair coefficient of variation (CV) between clusters is .058. The CV was calculated as CV = SQRT (ICC x (1 - phi) / phi), where the intraclass correlation coefficient (ICC) was assumed to be 0.01, and phi is 0.75 (i.e. the proportion of accuracy for one of the standard screening methods). The power will be slightly greater than 81% to detect values of 75% versus 85% in the AUC, based on power for comparing two dependent AUC's, assuming discrete cutoff values, and accounting for a sample inflation factor (IF), where IF = [1 + (M – 1) * ICC], M = average number of persons per cluster, and the ICC is 0.01.

Because the other studies are two separate pragmatic clinical trials, participants will be cluster-randomized to three different arms. This approach ensures that rigorous testing of the outcomes (incident ADRD detection and acceptance of subsequent diagnostic assessments) is not contaminated by the other screening approaches in other arms of interest. In addition, the 400 patients in study 1 will be excluded from the two pragmatic trials to avoid contamination. The total sample for each pragmatic trial will be slightly over 2,600, if each of enrolled practice screen conservatively 175 patients per year for a total of two years of screening. Alternatively, the total sample size for each trial will be 3,600, if we recruit, as we have in past trials, 200 patients per clinic. The table below shows the power for comparing ADRD incidence and acceptance rates between two different arms under the conservative and historical recruitment scenarios. Calculations were based on using nonlinear mixed models to compare two independent proportions, assuming cluster randomization (i.e., level-2 randomization) and the ICC is 0.01. The "AWV alone" arm is expected to have ADRD incidence of 6% and acceptance rate of 44%. The table shows that power will exceed 80% to detect ADRD incidence of 6% vs 13%, even under conservative recruitment projections, and power for comparing acceptance rates will be 99%.

Outcome	Conservative recruitment* (N = 2,600)	Projected (feasible historically) recruitment* (N = 3,600)
ADRD incidence 6% vs 13%	86%	89%
Acceptance 44% vs 66%	99%	99%

*Conservative recruitment = Total of 2,600 patients with 866 patients and 5 clusters per each of 3 arms; power for pairwise comparisons based on 1,732 patients with 866 patients and 5 clusters per each of 2 arms, and M = 173.2 patients per cluster. Historical recruitment = Total of 3,600 patients with 1,200 patients and 5 clusters per each of 3 arms; power for pairwise comparisons based on 2,400 patients with 1,200 patients and 5 clusters per each of 2 arms, and M = 240 patients per cluster.

D. 9. Potential problems & alternative strategies

First, while we expect that adding EHR-based data (the PMD) elements to the QDRS based process of ADRD detection will boost validity from 75% to 85% for AUC or for the overall % accuracy of classification, these gains may be marginal (<10%). Smaller gains would signal a precautionary lesson on the limits of EHRs to detect ADRD as currently designed. Second, given the heterogeneity of EHR data, adding EHR data might actually cause the ADRD detection process to gain validity at the cost of reliability. Given Medicare's growing reliance on EHRs for quality measurement, illuminating such a trade-off would be highly policy-relevant. Third, this work involves two states (Florida and Indiana), which may limit generalizability. However, the diversity of the study population may overcome these limitations. Fourth, the intervention faces barriers, such as stakeholder buy-in, unintended consequences, alert fatigue, and clinical uncertainty. We will overcome these barriers by (1) working closely with the leadership of the primary care practices to ensure stakeholder buy in, (2) leverage the trusted relationship between the patient and her primary care team, (3) the proposed PDM and QDRS will be fully integrated within the AWV and the clinical flow of the primary care practices, and (4) working with Drs. Grout and Dexter (their Epic Team) to trouble shoot EHR integration barriers and minimize alert fatigue. Based on our extensive preliminary studies, the combined approach of using the PDM and the QDRS would outperform the current state of using the AWV alone or the integration of the PDM within the AWV clinical flow. Fifth, seamlessly integrating the PDM and QRDS within the AWV clinical workflow with limited disruption can be challenging. The Regenstrief Institute has more than a decade of experience with, and has the technical infrastructure in place for, developing and integrating various CDS within Epic, Cerner, and other EHR vendors across the world (see Facilities & Other Resources). We have also allocated sufficient time between the analysis of Aim 1 and go-live of Aim 2 (see **Timetable**) to make any necessary adjustments. Lastly, we will be monitoring uptake and usage of the CDS intervention using the system logs. If uptake during the go-live period appears problematic, we will be able to revisit training / socializing the new workflow with clinical staff or alter the user interface based on feedback.

D.10. Timetable & Project Administration.

We are proposing a 5-year grant period to accommodate recruitment, management of multiple data sources, primary data collection, training data collection staff, and multiple distinct analytic techniques. During the first six months, we will hire and train our research team, set up the organizational structure of the research projects, and finalize IRB approval. In the following 18 months, we will deploy the PDM and the QDRS into the Epic EHR environments in the two health care systems in Indiana (Eskenazi Health) and Florida (UHealth). Also by the end of Year 2, we will recruit 400 subjects into our first clinical validation (Stage III) study and complete the data collection for Aim 1. In Year 3, we will complete the data analysis and the dissemination activities related to Primary and Secondary Aim 1. In the beginning of Year 3, we will recruit the

15 primary care practices from the two sites into our pragmatic cluster-randomized controlled comparative effectiveness (phase IV) trials. In Year 4 we will retrieve the data related to Primary Aim 2 from the Datawarehouse of the Epic EHR systems at both participating healthcare systems (Eskenazi Health and UHealth) and complete data analysis for Primary and Secondary Aim 2. In Year 5, we will share our de-identified data with the scientific community and disseminate the result of our trial to the scientific community via peer reviewed publication and presentation at national and international scientific conferences. In the same year, we will share our codes for both the PDM and the QDRS with Epic headquarters to make sure that these codes are available for any healthcare system with Epic across the country. We will also share technical details in a platform-agnostic manner to aid implementation in other EHR platforms.

Timetable

**Project Year**	**1**				**2**				**3**				**4**				**5**			
**Project Quarter**	**1**	**2**	**3**	**4**	**1**	**2**	**3**	**4**	**1**	**2**	**3**	**4**	**1**	**2**	**3**	**4**	**1**	**2**	**3**	**4**
Study start up	X	X																		
Deploy PDM* + QDRS** tools	X	X	X	X	X	X	X	X												
**Clinical Validation (stage III) Study**																				
Recruit subjects			X	X	X	X	X	X												
Data Collection (Aim 1)			X	X	X	X	X	X												
Data Analyses (Aim 1)									X	X										
Dissemination (Aim 1)											X	X								
**Pragmatic cluster-randomized controlled comparative effectiveness trials (phase IV)**																				
Recruit Clinics									X	X	X	X	X	X	X	X				
Data Retrieval from Epic (Aim 2)																	X	X		
Data Analysis (Aim 2)																	X	X		
Sharing Codes																	X	X	X	X
Sharing De-identified Data (Aim 2)																	X	X	X	X
Dissemination (Aim 2)																	X	X	X	X
*PDM: Passive Digital Marker; QDRS: Quick Dementia Rating Scale.																				

E. PROTECTION OF HUMAN SUBJECTS

Risk to Subjects

Human subjects' involvement and characteristics: Our Digital Detection of Dementia (**D**^{3}) project include the execution of three complimentary studies; the Clinical Validation (stage III) Study and the two Pragmatic cluster-randomized controlled comparative effectiveness (phase IV) trials. These studies will include community-dwelling men and women aged 65 years and older, who receive primary care services within Eskenazi Health located in central Indiana and UHealth in south Florida. They will be English or Spanish speaking, have had at least one visit to primary care practice within past year with available electronic health record (EHR) data from at least three years. The aims of the first Clinical Validation (Stage III) study require a face-to-face or telephone interview and EHR review, to evaluate the accuracy of two scalable approaches for early detection of Alzheimer's disease and related dementias (ADRD); the Passive Digital Marker (PDM) and the Quick Dementia Rating Scale (QDRS). These approaches will be embedded within the EHR systems of diverse rural, suburban, and urban primary care practices in central Indiana and south Florida. Older adult patients represent a somewhat vulnerable group. Our research specifically targets this group in an attempt to enhance the current standard of early detection of ADRD and improve Health for these individuals.

Participants in the first Clinical Validation Study will be informed of the purpose of the overall all goal of Digital Detection of Dementia (**D**^{3}) project of using scalable screening approached to improve the early detection of ADRD in primary care practices) and the procedures related to the clinical Validation study. Signed informed consent will be obtained from these participants. For the clinical validation study, and once informed consent is given, the research team will conduct a gold-standard ADRD assessments (within 30 days of screening) on all patients screened by the PDM and the QDRS.

Sources of material: We will collect EHR data from all patients enrolled in the Clinical Validation study (identified data after obtaining informed consent from subjects) and from patients receiving care in all of the practices enrolled in the Pragmatic cluster-randomized controlled comparative effectiveness (phase IV) trials (de-identified data after obtaining consent from the practice leadership and receiving waiver from the IRB as the two pragmatic trials are Quality improvement trials). For the clinical validation study, the research team will conduct a gold-standard ADRD assessments on all patients screened by the Passive Digital Marker and the Quick Dementia Rating Scale. Such ADRD assessment will be performed face-to-face at research centers in Indianapolis and Miami. To accommodate subject preference and current COVID-19 related safety, the ADRD assessment may be conducted via Zoom or at the subject's home. We have used such assessments for more than a decade with excellent quality assurance and completion. The ADRD assessment will be entered into a Redcap database.

Potential risks: No experimental pharmacological or nonpharmacological intervention will be used in this study. All patients enrolled in the clinical validation study will receive screening for ADRD to validate the accuracy of the PDM and the QDRS. Although data from this study will not be gathered from the patient without his or her consent, it is possible that some of the questions related to ADRD screening and management asked of the respondents could cause anxiety. However, in our recent published ADRD screening trial that evaluated the potential benefits and harms of ADRD screening, there were no differences between the screened and the control group in quality of life, depressive symptoms or anxiety.[17] Furthermore, we anticipate early detection of ADRD in some patients enrolled in the clinical validation study, and up to 13% of patients receiving care in all of the practices enrolled in the pragmatic trials who undergo the screening process and/or the subsequent diagnostic assessment. The impact of this early diagnosis on their quality of life and care was assessed in our recent published trial and found no negative impact on quality of life, depression, anxiety, healthcare utilization or independence

at home.[17] However, it is not known about other potential harms such as stigma. On the other hand, early detection of ADRD is considered part of the standard of care and screening for ADRD is part of the Annual Wellness Visit covered by Medicare. Such early detection would lead to receive appropriate and valuable care both pharmacologically and non-pharmacologically. Such treatment would decrease the patient's disability resulting from unrecognized or unmanaged ADRD and enhance the patients' adherence of medical management of other health conditions. The research personnel will be trained to recognize and minimize any potential discomfort and patients may discontinue participation at any time. If a respondent becomes anxious or upset, the local principal investigator will be notified to intervene. Loss of confidentiality is also a risk in this type of data collection. Our data management and quality assurance technique has proven effective in past trials in maintaining confidentiality, and all study personnel have completed training in Human Subjects Research and HIPAA standards.

Adequacy of Protection Against Risks

Recruitment and informed consent: The Digital Detection of Dementia (**D³**) studies will be approved by the Indiana University-Purdue University at Indianapolis Institutional Review Board. Indiana University-Purdue University (IUPUI) at Indianapolis Institutional Review Board will be the single IRB for this trial. University of Miami will submit a request for a reliance agreement. Trained research assistants will work with the practice managers from each primary care practice to identify potential subjects who meet the inclusion and exclusion criteria for the Clinical Validation study. Recruitment will be performed over the telephone, Zoom, or in-person at primary care practices for those eligible to participate in the clinical validation study, following HIPAA compliant standard recruitment procedures. The research assistants will make initial contact via telephone, Zoom or face to face at the primary care practices to garner interest in participating in the study and verifying the patients' eligibility. Our studies will require two written informed consent forms. The first one, for the clinical validation study will be completed per eligible participant and countersigned by the researcher and the second one, is consent from the practice leadership, after receiving an informed consent waiver from the IRB for patients receiving care in all of practices enrolled in the pragmatic trials. The informed consent process will be completed prior to conducting any baseline assessments.

Protection against risk: Patients who refuse to participate in the Clinical Validation study will not be approached again. The patient's medical record will only be reviewed after obtaining informed consent from the participants in the clinical validation study (Stage III) and from the practice leadership (after receiving an informed consent waiver from the IRB), for patients receiving care in all of practices enrolled in the pragmatic trials (Phase IV). To avoid the risk of inadvertent disclosure of PHI, all study personnel will be educated about general principles of subject access, informed consent, confidentiality, data safeguarding, and privacy requirements, and will complete an on-line course required by the Indiana University Purdue University Institutional Review Board. The format and the procedure of data collection will be outlined in the consent form, the data will be extracted into a pre-designed database, and the research assistant will not collect any additional data without the consent of the patient and approval by university IRB. Each study participant will be assigned a study identification (ID) number, and this ID number will be used rather than names and medical record numbers on all PHI data collected.

The principal investigators will disclose the finding of new information about the patient's health status directly to the patient and if the participant agrees, to the primary care physician. The principal investigators will be available to clarify any questions and offer any needed consultation. Efforts will be made to keep patients' personal information confidential. In addition,

the investigators are planning to have continuous feedback from the patient and the practice staff to discuss any problems encountered during the data collection.
Reporting unanticipated problems involving risk to participants: A data safety monitoring board (**DSMB**) will be established, and will include a primary care physician, Geriatrician, patient representative, and biostatistician. The DSMB will meet prior to the first patient enrollment, to review the protocol for any major concerns prior to implementation. Subsequently, the DSMB will meet periodically to review and evaluate the accumulated study data for participant safety, study conduct and progress and scientific validity and integrity of the trial.

Potential Benefits of the Proposed Research to the Subjects and Others
Although our studies are collecting data to test the accuracy and effectiveness of two scalable approaches for early detection of (ADRD) among older adults attending primary care practices, we anticipate some immediate benefit to participants. Detecting cognitive decline early and at low-cost is beneficial to patients and caregivers alike. The screening results will assure of the negative screening results, and those with positive screening results will receive a referral for appropriate diagnostic and management services. Early detection of ADRD will lead to early management of its cognitive, functional, and psychological disabilities, which if goes unmanaged, results in significant burden for patients, families, healthcare delivery systems and the entire society. In turn, early detection of ADRD could reduce such a burden, in addition to becoming increasingly important for assuring that people receive early ADRD disease modification treatment when available.

Importance of the Knowledge to be Gained
Alzheimer's disease and related dementias (ADRD) negatively impact millions of Americans. Currently, half of Americans living with ADRD never receive a diagnosis. For those who do, the diagnosis often occurs two to five years after the onset of symptoms. The data may lead to national early detection and management program for ADRD as an efficient and beneficial method of reducing the current and future burden of ADRD. We are unaware of any similar studies that has been or will be conducted in a diverse primary care setting. Our studies will inform the scientific community and the health care system about the performance, including accuracy and effectiveness of the two scalable approaches in early detection of ADRD and improving the annual rate of new documented ADRD in primary care practices. Our studies will provide some knowledge about the specific characteristics of the primary care patients, and their attitudes toward early detection of ADRD and subsequent referral for appropriate diagnostic and management services. The knowledge gained will increase the power of care delivery at our study sites by leveraging machine learning. This will make our health systems smarter and help to prevent avoidable hospitalizations and other negative ADRD outcomes.

ClinicalTrials.gov Registration: The Digital Detection of Dementia (**D^3**) studies will be registered at clinicaltrials.gov.

F. DATA AND SAFETY MONITORING PLAN

The proposed pragmatic Clustered-comparative effectiveness trials are considered Quality Improvement project. However, we will construct a data safety monitoring plan (DSMP) for the trials and the clinical validation study. This DSMP will be monitored by the co-PIs and a three-member Data Safety and Monitoring Board (DSMB). The co-PIs will also conduct data and safety monitoring and will regularly monitor progress, goal achievement, and overall research direction in consultation with the co-investigators. In regard to the pragamtic trials, the team will use the Epic EHR system log to monitor the uptake and usage of the Annual Wellness visit with and without the deployed screening approaches of the Passive Digital Marker (PDM) and the Quick Dementia Rating Scale (QDRS) within the Epic computerized decision support (CDS). If uptake during the go-live period appears problematic, we will be able to revisit training and socializing the enhanced AWV with clinical staff or alter the user interface based on feedback.

Data Monitoring and Reporting

The frequency of data review for this studies are summarized in the following tables:

**Data Type: Clinical Validation Study**	Frequency of Review		
	Each Occurrence	At assessment (Baseline)	End of study
Subject accrual (adherence to inclusion/exclusion); completion of the screening and the diagnostic assessment		X	
Protocol violations/noncompliance	X		
Out of range data		X	
Risk-benefit ratio assessment			X

**Data Type: Pragmatic Trials– All data capture will be from the EHR**		
	Quarterly	Annual
Adverse events, Unscheduled acute care utilization	X	X
Usage of the AWV with and without the PDM and the QDRS	X	X
Risk- Benefit ratio assessment		X

The project manager and the biostastician will generate reports for each pragmatic trial that will contain:

A) summary of all-source adverse events and an explanation of how each event was handled;
B) the usage of the AWV with and without the PDM or the QDRS, and
C) completion of the diagnostic assessment for those who screened positive;

All reports will be submitted to the Indiana University Purdue University of Indianapolis (IUPUI) IRB at time of continuing review.
Note: University of Miami will submit a request for a reliance agreement; IUPUI IRB will be the reviewing IRB for this study.

Protection of data

Database security is maintained using a multi-layered approach to both limit access and the ability to alter data. There are strong protections restricting access to the researchers'

network. Local installation of proprietary software is required to detect our server on the network. Each authorized user has a unique username and password that allows specific access and editing privileges. Browsing level access only is used except when data entry/editing is explicitly intended. While multiple authorized users can simultaneously view data, only a single specifically authorized user can edit a given record at any time. The database is stored on a secure server, which is also password protected and only accessible to authorized lab personnel, in our locked laboratory offices. Database backups are conducted on a regular basis. Raw data, regardless of capture method, are accessible only to key research personnel; and all electronic information is stored on password-protected computers. The PIs will additionally regularly monitor the data collection and analysis process for data appropriateness, comprehensiveness, accuracy, and timeliness.

Safety Monitoring Definitions

Adverse Event (AE) - Any untoward or unfavorable medical occurrence in a human subject, including any abnormal sign, symptom, or disease, temporally associated with the subject's participation in the research, whether or not considered related to the subject's participation in the research. We will use the EHR system to monitor the following AEs:

- Emergency room visits, not requiring hospitalization
- Breach of confidentiality
- Suicidal ideation not requiring intervention

Serious Adverse Event (SAE) is any adverse events that result in the following, which we will monitor:

- Death
- Persistent or significant disability/incapacity
- Inpatient hospitalization or prolongation of existing hospitalization
- Suicidal attempt or ideation requiring intervention

Study-Related – An AE or SAE is considered study related if the PI determines that the AE or SAE to be definitely, probably, or possibly related where possibly related means there is reasonable possibility that the incident, experience, or outcome may have been caused by the procedures or interventions involved in the research. AEs or SAEs judged as remotely related or not related are not considered study related.

Unanticipated Problem or Unexpected AE/SAE – problems or events in which the research places participants or others at a greater risk of harm (including physical, psychological, economic, or social harm) than was previously known or recognized. The term “unanticipated problem” is used in this context because some situations may not have produced an adverse event but is still considered an unanticipated problem (e.g. unsecured or stolen patient data which may not result in an AE). An event is unexpected if it is not described in the package insert of cessation medication, in the study protocol, or in the informed consent document.

Measurement and Reporting of Adverse Events

Adverse event rates associated with screening interventions are low and are expected to vary little between the intervention and control groups in the two pragmatic trials. We will present blinded adverse event data to the co-PIs throughout the trials. We plan to present unblinded adverse events data to the DSMB panel when requested and at the annual meetings. If there is evidence of elevated adverse events, the PIs will consult with the

study team and use an adverse event form to report injuries or other adverse events caused by the intervention and detected through sources listed below.

Event Detection and Reporting

No serious adverse events (SAEs) are expected for the two pragmatic trials. Our CHOICE trial[17] found no negative impact of screening on depression, anxiety, quality of life, or health care utilization; thus, the only expected Adverse Events (AE) is loss of confidentiality. The Annual Wellness visit is the current standard of care.

All AEs reported by any of the primary care clinics or detected by the quarterly EHR review will be recorded and monitored. Each event will be documented on the basis of AE or SAE, severity, expectancy, and relatedness (definitely related, possibly related, or not related to study activities). The Principal Investigators (PIs) will be notified of all reportable events, including SAEs, within 24 hours. SAEs will be reported by telephone to the IUPUI IRB within 48 hours. A summary of SAEs will be included in annual IRB reporting. Annual Reports submitted to the IRB will contain the number of AEs and SAEs in the prior year and an explanation of how each event was handled. The annual IRB report will also contain the following: number of complaints and how each complaint was handled and the number of patient who did not complete the diagnostic assessment following screen positive results within the AWV.
SAEs that are unanticipated and definitely related to the intervention will be reported to the NIH Program Officer within 48 hours of the study's knowledge of the SAE.

<u>SAE/AE Response and Reporting Procedure</u>

1) Research staff becomes aware of AE/SAE (via scheduled reviews of EHR data)
2) Research staff notifies Dr. Boustani and/or Dr. Galvin immediately if the event is an SAE or if immediate psychiatric or medical intervention is required, and within 7 days if the event is an AE.
3) Dr. Boustani and/or Dr. Galvin will conduct EHR review when necessary to gather additional information about the event.
4) Dr. Boustani and/or Dr. Galvin will report AE/SAE to DSMB to aid in determining:
    a. <u>Severity</u>: Mild, Moderate, Severe
    b. <u>Expectedness</u>: Expected, Unexpected
    c. <u>Study Related</u>: Definitely, Probably, Possibly, Remotely, Note Study-Related
5) Project staff documents AE/SAE and DSMB determination in study database. The report will be provided to a NIA Program Officer.
6) Dr. Boustani and/or Dr. Galvin will prepare a report for the IRB as per local, state, and federal reporting requirements.
7) If a death occurs, Dr. Boustani and/or Dr. Galvin will report it to the DSMB and NIA Program Officer within 48 hours of knowing about the event.
8) DSMB will aid in planning measures to prevent future occurrences, if any warranted.
9) Dr. Boustani and/or Dr. Galvin will make changes to protocol and/or consent form if needed.

To enhance monitoring and study oversight, DSMB and study staff will meet once per 6 month.

Possible Adverse Events

Adverse events will be monitored on an ongoing basis by the study manager from three sources:

1. Self-initiated phone, e-mail, or in-person report by the participant in the clinic al validation study or by the primary care clinical team for the pragmatic trials.

2. Quarterly review of acute care utilization in the pragmatic trials.

The co-PIs will be notified within 24 hours of any adverse events. Serious adverse events will be reported within 5 business days to IUPUI IRB, DSMB, and NIH. Non-serious adverse events will be reported at time of continuing review to IUPUI IRB. In cases where there is any question regarding the level of adverse event or attributable cause, we will consult with the DSMB.

Non-serious adverse events are defined as: 1) outpatient surgical procedure, 2) sought advice from a physician or medical professional regarding a problem related to prescription medications, 3) experienced pain, fatigue, dizziness, or acute illness requiring no hospitalization related to prescription medication use. Serious adverse events are defined as: death, life threatening event, inpatient hospitalization, permanent disability.

Stopping Rules
The two pragmatic trials are quality improvement studies of clinical process related to the operationalization of the Annual Wellness visit that is covered by Medicare for the sole purpose of improving the recognition of ADRD by the healthcare system. It is unlikely that the trial would be stopped early. The NIH will make the final decision on whether or not to accept the DSMB's recommendation about discontinuation of any component of the study.

Limits of Assumptions
It is possible that baseline differences between the groups or missing data will limit the value of data analysis of measurements. Baseline differences will be evaluated after the baseline measurement time point at the halfway point of target enrollment. Effects on the power to detect differences in the primary outcome will be evaluated and communicated to the PI, DSMB, and NIH. Given the monitoring plans outlined elsewhere in this document, it is exceedingly unlikely that there will be baseline differences between groups of any magnitude to threaten the validity of the studies.
With early alerts to problems, action would be taken to avoid higher level alerts; if a higher level alert should arise, more drastic remedial action would be invoked.
The actions taken at each level of alert are given below:

- Mid-level alert = Conference call between study investigators to discuss approaches to minimize further losses to follow-up/dropouts.
- High-level alert = Conference call between investigators and DSMB to determine further alterations of study protocol to complete the study with no further losses
- Extreme-level alert = In the unlikely event of a 45% dropout rate occurs, study investigators, the DSMB members, and the NIH program official would convene on a conference call to discuss the usefulness of continuing the study.

Qualifications and Responsibilities of the Data Safety and Monitoring Board (DSMB)
The three-member DSMB panel will meet at 6-month intervals and has the following responsibilities:

- Review the research protocol for the three studies, informed consent documents of the clinical validation study and plans for data safety and monitoring for the two pragmatic trials;
- Initiate studies after having deemed the protocols are satisfactory;
- Evaluate the progress of the three studies including periodic assessments of data quality and timeliness, recruitment, participant risk versus benefit, performance of the study site, and other factors that can affect study outcomes;

- Consider factors external to the study when relevant information becomes available, such as scientific or therapeutic developments that may have an impact on the safety of the participants or the ethics of the study;
- Review study performance, resolve problems with study operations;
- Protect the safety of the study participants;
- Report to NIH on the safety and progress of the trial;
- Confer with NIH Program Officer concerning continuation, termination or other modifications of the study based on adverse events;
- Ensure the confidentiality of the study data and the results of monitoring; and,
- Communicate with NIH regarding any problems with study conduct, enrollment, sample size, and/or data collection.

DSMB Membership and Affiliation

We will identify the members of the DSMB in collaboration with the National Institute on Aging. These persons will not be otherwise affiliated with the project. Should there be any questions regarding the independence of the DSMB, it will be addressed and corrected, if necessary, at that time.

The following individuals would be recommended to the NIH.

Jim Rudolph, MD
Director, Center of Innovation in Geriatric Services, Providence VA Medical Center
Professor of Medicine and Health Policy & Practice, The Warren Alpert Medical School of Brown University

Esther Oh, MD, PhD
Associate Director, Johns Hopkins Memory and Alzheimer's Treatment Center
Associate Professor of Medicine, Johns Hopkins

Manish Shah, MD, MPH
Professor and Vice Chair of Research, University of Wisconsin-Madison School of Medicine and Public Health
The John & Tashia Morgridge Chair of Emergency Medicine Research
Director/KL2 Program, Institute for Clinical and Translational Research
Co-Lead, Care Research Core, Alzheimer's Disease Research Center

<u>Conflict of Interest for DSMB's</u>

DSMB should have no direct involvement with the study investigators or intervention. Each DSMB member will sign a Conflict of Interest Statement which includes current affiliations, if any, with pharmaceutical and biotechnology companies (e.g., stockholder, consultant), and any other relationship that could be perceived as a conflict of interest related to the study and/or associated with commercial interest pertinent to study objectives.

<u>Protection of Confidentiality</u>

Data with PHI will only be shared with members of the research team that are authorized to view such data as part of research procedures. De-identified data will be presented at open sessions of DSMB meetings. Participants' identities will not be known to the DSMB members and all data will be treated as confidential.

References

1. Alzheimer's Association. 2018 Alzheimer's Disease Facts and Figures. 2019; https://www.alz.org/media/Documents/alzheimers-facts-and-figures-2019-r.pdf. Accessed 2020 Jan 17.
2. Cordell CB, Borson S, Boustani M, et al. Alzheimer's Association recommendations for operationalizing the detection of cognitive impairment during the Medicare Annual Wellness Visit in a primary care setting. Alzheimers Dement. 2013; 9:141-150.
3. Holsinger T, Deveau J, Boustani M, Williams JW. Does this patient have dementia? JAMA 2007; 297(21):2391-404.
4. Bradford A, Kunik ME, Schulz P, Williams SP, Singh H. Missed and delayed diagnosis of dementia in primary care: prevalence and contributing factors. Alzheimer Dis Assoc Disord. 2009; 23: 306-314.
5. Lin JS, O'Connor E, Rossom RC, Perdue LA, Eckstrom E. Screening for cognitive impairment in older adults: A systematic review for the U.S. Preventive Services Task Force. Ann Intern Med. 2013; 159: 601- 612.
6. Boise L, Camicioli R, Morgan DL, Rose JH, Congleton L. Diagnosing dementia: perspectives of primary care physicians. Gerontologist. 1999; 39:457-464.
7. Brayne C, Fox C, Boustani M. Dementia screening in primary care: is it time? JAMA. 2007; 298:2409-2411.
8. Iliffe S, Haines A, Gallivan S, Booroff A, Goldenberg E, Morgan P. Assessment of elderly people in general practice. 1. Social circumstances and mental state. Br J Gen Pract. 1991; 41:9-12.
9. McCormick WC, Kukull WA, van Belle G, Bowen JD, Teri L, Larson EB. Symptom patterns and comorbidity in the early stages of Alzheimer's disease. J Am Geriatr Soc. 1994; 42:517-521.
10. Ben Miled, Z., Haas, K., Black, C. M., Khandker, R. K., Chandrasekaran, V., Lipton, R., & Boustani, M. A. (2019). Predicting dementia with routine care EMR data. Artificial Intelligence in Medicine. 2020 January; 102:101771.
11. Galvin JE. The Quick Dementia Rating System (QDRS): A rapid dementia staging tool. Alzheimer Dem (DADM) 2015; 1:249-259.
12. Boustani M, Callahan CM, Unverzagt FW, et al. Implementing a screening and diagnosis program for dementia in primary care. JGIM. 2005; 20(7): 572-7 doi: 10.1111/j.1525-1497.2005.0126.x.
13. Ashford JW, Borson S, O'Hara R, et al. Should older adults be screened for dementia? It is important to screen for evidence of dementia! Alzheimers Dement. 2007; 3:75-80.
14. Hurd MD, Martorell P, Delavande A, Mullen KJ, Langa KM. Monetary costs of dementia in the United States. N Engl J Med. Apr 04 2013; 368(14):1326-1334.
15. Callahan CM, Boustani MA, Unverzagt FW, et al. Effectiveness of collaborative care for older adults with Alzheimer disease in primary care: a randomized controlled trial. JAMA. 2006; 295: 2148-2157.
16. Boustani M, Alder C, Solid C, Reuben D. An alternative payment model to support widespread use of collaborative dementia care models. Health Affairs. 2019; 38(1):54-59.
17. Fowler NR, Perkins AJ, Gao S, Sachs GA, and Boustani MA. Risks and Benefits of Screening for Dementia in Primary Care: The Indiana University Cognitive Health Outcomes Investigation of the Comparative Effectiveness of Dementia Screening (IU CHOICE) Trial. J Am Geriatr Soc. 2019; 00:1-9 (published online Dec 2 2019; DOI:10.1111/jgs.16247).

18. The ABCs of Annual Wellness Visit (AWV). Available at: https://www.cms.gov/Outreach-and-Education/Medicare-Learning-Network-MLN/MLNProducts/downloads/AWV_chart_ICN905706.pdf. Accessed Jan 17, 2020.
19. Fowler NR, Campbell NL, Pohl GM, Munsie LM, Kirson NY, Desai U, Trieschman EJ, Meiselbach MK, Andrews JS, Boustani MA. One-Year Effect of the Medicare Annual Wellness Visit on Detection of Cognitive Impairment: A Cohort Study. J Am Geriatr Soc. 2018 May; 66(5):969-975. doi: 10.1111/jgs.15330. Epub 2018 Apr 2. PubMed PMID: 29608782.
20. US Department of Health and Human Services. National Plan to Address Alzheimer's Disease: 2018 Update. 2018; https://aspe.hhs.gov/system/files/pdf/259581/NatPlan2018.pdf. Accessed 22 Mar 2019.
21. Reuben DB, Hackbarth AS, Wenger NS, Tan ZS, Jennings LA. An Automated Approach to Identifying Patients with Dementia Using Electronic Medical Records. J Am Geriatr Soc. Mar 2017; 65(3):658-659.
22. Jammeh EA, Carroll CB, Pearson SW, et al. Machine-learning based identification of undiagnosed dementia in primary care: a feasibility study. BJGP Open. Jul 2018; 2(2):bjgpopen18X101589.
23. Amra S, O'Horo JC, Singh TD, et al. Derivation and validation of the automated search algorithms to identify cognitive impairment and dementia in electronic health records. J Crit Care. Feb 2017; 37:202-205.
24. Maity NG and Das S. Machine learning for improved diagnosis and prognosis in healthcare. IEEE Aerospace Confernece. 2017. DOI: 10.1109/AERO.2017.7943950.
25. Philpis E, Walters A, Biju M, Kuruvilla T. Population-based screening for dementia: controversy and current status. Progress in Neurology and Psychiatry. 2016; Jan/Feb:6-10.
26. Galvin JE. Using informant and performance screening methods to detect mild cognitive impairment and dementia. Curr Geriatr Rep. 2018 Mar; 7(1):19-25.
27. Fowler NR, Perkins AJ, Turchan HA, et al. Older primary care patients' attitudes and willingness to screen for dementia. J Aging Res. 2015; 2015:423265.
28. Harrawood A, Fowler NR, Perkins AJ, LaMantia MA, Boustani MA. Acceptability and Results of Dementia Screening Among Older Adults in the United States. Curr Alzheimer Res. 2018; 15(1): 51-55.
29. Fowler NR, Perkins AJ, Gao S, Sachs GA, Uebelhor AK, Boustani MA. Patient characteristics associated with screening positive for Alzheimer's disease and related dementia. Clin Interv Aging. 2018;13:1779-1785.
30. Boustani MA, Justiss MD, Frame A, et al. Caregiver and noncaregiver attitudes toward dementia screening. J Am Geriatr Soc. Apr 2011; 59(4):681-686.
31. Fowler NR, Boustani MA, Frame A, et al. Effect of patient perceptions on dementia screening in primary care. J Am Geriatr Soc. Jun 2012; 60(6): 1037-1043.
32. Justiss MD, Boustani M, Fox C, et al. Patients' attitudes of dementia screening across the Atlantic. Int J Geriatr Psychiatry. Jun 2009; 24(6):632-637.
33. Monahan PO, Alder CA, Khan BA, Stump T, Boustani MA. The Healthy Aging Brain Care (HABC) monitor: validation of the patient self-report version of the clinical tool designed to measure and monitor cognitive, functional, and psychological health. Clin Interv Aging. 2014; 9:2123-32 doi: 10.2147/cia.S64140.
34. Holmes MM, Stanescu S, Bishop FL. The use of measurement systems to support patient self-management of long term conditions: an overview opportunities and challenges. Patient Relat Outcome Meas. 2019 Dec 16; 10:385-394.
35. Boulesteix AL, Janitza S, Kruppa J, Konig IR. Overview of random forest methodology and practical guidance with emphasis on computational biology and bioinformatics. Wiley Interdisciplinary Reviews: Data Mining and Knowledge Discovery. Vol 2 2012:493-507.

36. Seixas FL, Zadrozny B, Laks J, Conci A, Muchaluat Saade DC. A Bayesian network decision model for supporting the diagnosis of dementia, Alzheimers disease and mild cognitive impairment. Comput Biol Med. Aug 2014; 51140-158.

37. Zhang D, Shen D. Multi-modal multi-task learning for joint prediction of multiple regression and classification variables in Alzheimer's disease. Neuroimage. Jan 16 2012; 59(2):895-907.

38. Moradi E, Pepe A, Gaser C, Huttunen H, Tohka J. Machine learning framework for early MRI-based Alzheimer's conversion prediction in MCI subjects. Neuroimage. Jan 1 2015; 104:398-412.

39. Zhang Y, Dong Z, Phillips P, et al. Detection of subjects and brain regions related to Alzheimer's disease using 3D MRI scans based on eigenbrain and machine learning. Front Comput Neurosci. 2015; 9:66.

40. Chen R, Herskovits EH. Machine-learning techniques for building a diagnostic model for very mild dementia. Neuroimage. Aug 1 2010; 52(1):234-244.

41. Shankle WR, Mani S, Pazzani MJ, Smyth P. Detecting very early stages of dementia from normal aging with machine learning methods. Paper presented at: Conference on Artificial Intelligence in Medicine1997.

42. Maroco J, Silva D, Rodrigues A, Guerreiro M, Santana I, de Mendonca A. Data mining methods in the prediction of Dementia: A real-data comparison of the accuracy, sensitivity and specificity of linear discriminant analysis, logistic regression, neural networks, support vector machines, classification trees and random forests. BMC Res Notes. Aug 17 2011; 4:299.

43. Woods SP, Troster AI. Prodromal frontal/executive dysfunction predicts incident dementia in Parkinson's disease. J Int Neuropsychol Soc. Jan 2003; 9(1):17-24.

44. Gianattasio KZ, Wu Q, Glymour MM, and Power MC. Comparison of Methods for Algorithmic Classification of Dementia Status in the Health and Retirement Study. Epidemiology. 2019; 30:291–302.

45. Miotto R, Li L, Kidd BA, Dudley JT, Deep patient: an unsupervised representation to predict the future of patients from the electronic health records. Sci Rep. 2016 May 17; 6:2609.

46. Buschke H, Kuslansky G, Katz M, et al. Screening for dementia with the memory impairment screen. Neurology. Jan 15 1999;52(2):231-238.

47. Lipton RB, Katz MJ, Kuslansky G, et al. Screening for dementia by telephone using the memory impairment screen. J Am Geriatr Soc. Oct 2003;51(10):1382-1390.

48. Borson S, Scanlan J, Brush M, Vitaliano P, Domak A. The mini-cog: a cognitive 'vital signs' measure for dementia screening in multi-lingual elderly. Int J Geriatr Psychiatry. 2000; 15(11):1021-7.

49. Horsman J, Furlong W, Feeny D, Torrance G. The Health Utilities Index (HUI): concepts, measurement properties and applications. Health Qual Life Outcomes. 2003; 1:54.

50. Kroenke K, Spitzer RL. The PHQ-9: A new depression and diagnostic severity measure. Psychiatric Annals. 2002; 32:509-521.

51. Spitzer RL, Kroenke K, Williams JB, Lowe B. A brief measure for assessing generalized anxiety disorder: the GAD-7. Arch Intern Med. 2006; 166:1092-1097.

52. Boustani M, Perkins AJ, Khandker RK, Duong S, Dexter PR, Lipton R, Black CM, Chandrasekaran V, Solid CA, and Monahan P. Passive Digital Signature for Early Identification of Alzheimer’s Disease and Related Dementia. J Am Geriatr Soc. 2019; XX: 1-8 (published online on Nov 29 2019);
DOI:10.1111/jgs.16218.)

53. Galvin JE, Roe CM, Powlishta KK et al. The AD8: a brief informant interview to detect dementia. Neurology. 2005; 65:559–594.

54. Galvin JE, Roe CM, Xiong C, Morris JC. The validity and reliability of the AD8 informant interview for dementia. Neurology. 2006; 67:1942-1948.

55. Berman SE, Koscik RL, Clark LR, Mueller KD, Bluder L, Galvin JE, Johnson SC. Use of the Quick Dementia Rating System (QDRS) in the Wisconsin Registry for Alzheimer’s Prevention. J Alz Dis Report. 2017; 1:9-13.

56. Boustani M, Yourman L, Holden RJ, Pang PS, Solid, CA. A profile in population management: The Sandra Eskenazi Center for Brain Care Innovation. Generations Supplement. 2019; 3:68-72.

57. Holden RJ, Campbell NL, Abebe E, Clark DO, Ferguson D, Bodke K, Boustani MA, Callahan CM, Brain Health Patient Safety Laboratory. Usability and feasibility of consumer-facing technology to reduce unsafe medication use by older adults. RSAP 2019. doi: 10.1016/j.sapharm.2019.02.011. Epub 2019 Feb 26. (In Press)

58. Khan BA, Perkins AJ, Campbell NL, Gao S, Farber MO, Wang S, Khan SH, Zarzaur BL, Boustani MA. Pharmacological management of delirium in the intensive care unit: a randomized pragmatic clinical trial. J Am Geriatr Soc. 2019 May; 67(5):1057-1065.

59. Campbell NL, Perkins AJ, Khan BA, Gao S, Farber MO, Khan S, Wang S, Boustani MA. Deprescribing in the pharmacologic management of delirium: a randomized trial in the intensive care unit. J Am Geriatr Soc. 2019; 67(4):695-702. doi: 10.1111/jgs.15751.

60. LaMantia MA, Perkins AJ, Gao S, Austrom MG, Alder CA, French DD, Litzelman DK, Cottingham AH, Boustani MA. Response to depression treatment in the Aging Brain Care Medical Home model. Clin Interv Aging. 2016; 11:1551-58 doi: 10.2147/cia.S109114.

61. LaMantia MA, Alder CA, Callahan CM, Gao S, French DD, Austrom MG, Boustany K, Livin L, Bynagari B, Boustani MA. The Aging Brain Care Medical Home: Preliminary Data. J Am Geriatr Soc. 2015;
63(6):1209-13 doi: 10.1111/jgs.13447.

62. Khan BA, Calvo-Ayala E, Campbell N, Perkins A, Ionescu R, Tricker J, Campbell T, Zawahiri M, Buckley JD, Farber MO, Boustani MA. Clinical decision support system and incidence of delirium in cognitively impaired older adults transferred to intensive care. Am J Crit Care. 2013; 22(3):257-62 doi: 10.4037/ajcc2013447.

63. Frame A, LaMantia M, Reddy Bynagari BB, Dexter P, Boustani M. Development and implementation of an electronic decision support to manage the health of a high-risk population: the enhanced Electronic Medical Record Aging Brain Care software (eMR-ABC). EGEMS (Washington, DC). 2013; 1(1):1009 doi: 10.13063/2327-9214.1009.

64. Boustani MA, Campbell NL, Khan BA, et al. Enhancing care for hospitalized older adults with cognitive impairment: a randomized controlled trial. JGIM. 2012; 27(5):561-7 doi: 10.1007/s11606-012-1994-8.

65. Kasthurirathne SN, Vest J, Menachemi N, Halverson PK, Grannis SJ. Assessing the capacity of social determinants of health data to augment predictive models identifying patients in need of wraparound social services. J Am Med Informatics Assoc. 2018;25(1). doi:10.1093/jamia/ocx130

66. Haas LR, Takahashi PY, Shah ND, Stroebel RJ, Bernard ME, Finnie DM, Naessens JM. Risk-stratification methods for identifying patients for care coordination. Am J Manag Care. 2013; 19(9):725–
32. PMID: 24304255.

67. Vest JR, Menachemi N, Grannis SJ, Ferrell JL, Kasthurirathne SN, Zhang Y, Tong Y, Halverson PK. Impact of Risk Stratification on Referrals and Uptake of Wraparound Services That Address Social Determinants: A Stepped Wedged Trial. Am J Prev Med. 2019 Feb 14; 56(4):e125–e133.
doi:10.1016/j.amepre.2018.11.009 PMID: 30772150.

68. Beekly DL, Ramos EM, Lee WW, Deitrich WD, Jacka ME, Wu J, Hubbard JL, Koepsell TD, Morris JC, Kukull WA; NIA Alzheimer's Disease Centers. The National Alzheimer's Coordinating Center (NACC) database: the Uniform Data Set. Alzheimer Dis Assoc Disord. 2007 Jul-Sep; 21(3):249-58.

69. Weintraub S, Besser L, Dodge HH, Teylan M, Ferris S, Goldstein FC, Giordani B, Kramer J, Loewenstein D, Marson D, Mungas D, Salmon D, Welsh-Bohmer K, Zhou XH, Shirk SD, Atri A, Kukull WA, Phelps C, Morris JC. Version 3 of the Alzheimer Disease Centers' Neuropsychological Test Battery in the Uniform Data Set (UDS). Alzheimer Dis Assoc Disord. 2018 Jan-Mar; 32(1):10-17.
70. Nasreddine ZS, Phillips NA, Bédirian V, Charbonneau S, Whitehead V, Collin I, Cummings JL, Chertkow H. The Montreal Cognitive Assessment, MoCA: a brief screening tool for mild cognitive impairment. J Am Geriatr Soc. 2005 Apr; 53(4):695-9.
71. Shapiro AM, Benedict RH, Schretlen D, Brandt J. Construct and concurrent validity of the HopkinsVerbal Learning Test-revised. Clin Neuropsychol. 1999 Aug; 13(3):348-58.
72. Reitan, R.M., Validity of the trail making test as an indication of organic brain damage. Perceptual and Motor Skills. 1958; 8:271-276.
73. Morris JC. The Clinical Dementia Rating (CDR): Current version and scoring rules. Neurol. 1993; 43:2412-2414.
74. Kaufer DI, Cummings JL, Ketchel P, Smith V, MacMillan A, Shelley T, Lopez OL, DeKosky ST. Validation of theNPI-Q, a brief clinical form of the Neuropsychiatric Inventory. J Neuropsychiatry Clin Neurosci. 2000 Spring; 12(2):233-9.
75. Tappen RM, Rosselli M, Engstrom G. Evaluation of the Functional Activities Questionnaire (FAQ) in cognitive screening across four American ethnic groups. Clin Neuropsychol. 2010 May; 24(4):646-61.
76. Callahan CM, Boustani MA, Schmid AA, et al. Targeting functional decline in Alzheimer disease: a randomized trial. Annals of internal medicine. 2017; 166(3):164-71 doi: 10.7326/m16-0830.
77. Kim e, Zhang Z, Wang Y, Zeng D. Power calculation for comparing diagnostic accuracies in a multi-reader, multi-test design. Biometrics. 2014; 70(4):1033-41. doi: 10.1111/biom.12240. Epub 2014 Oct 29.
78. Obuchowski NA. Nonparametric analysis of clustered ROC curve data. Biometrics. 1997; 53(2):567-78.
79. DeLong, E. R., DeLong, D. M., and Clarke-Pearson, D. L.Comparing the areas under two or more correlated receiver operating characteristic curves: A nonparametric approach. Biometrics. 1988; 44:837-844.
80. Rao, J. N. K. and Scott, A. J. A simple method for the analysis of clustered binary data. Biometrics. 1992; 48:577-585.
81. Little RJ, D'Agostino R, Cohen ML, Dickersin K, Emerson SS, Farrar JT, Frangakis C, Hogan JW, Molenberghs G, Murphy SA, Neaton JD, Rotnitzky A, Scharfstein D, Shih WJ, Siegel JP, Stern H. The prevention and treatment of missing data in clinical trials. N Engl J Med. 2012 Oct 4; 367(14):1355-60.
82. PASS 2019 Power Analysis and Sample Size Software (2019). NCSS, LLC. Kaysville, Utah, USA, ncss.com/software/pass.
