# Supplementary material for: Digital Detection of Dementia in Primary Care: A Randomized Clinical Trial
Source: JAMA Netw Open. 2025 Nov 10;8(11):e2542222. doi: 10.1001/jamanetworkopen.2025.42222 (PMC12603861; doi:10.1001/jamanetworkopen.2025.42222)
Supplement: Supplement 2. — eFigure 1. Cumulative Incidence of Completed ADRD Diagnostic Assessments by Study Arm During a 12-Month Follow-Up eFigure 2. Cumulative Incidence of Completed ADRD Diagnostic Assessments by Study Arm During a 12-Month Follow-Up Period by Type of Diagnostic Assessment eAppendix. The Computerized Decision Support Workflow and Display eFigure 3. The In-Basket Message to the Primary Care Clinician eTable 1. Intervention Effect on the Cumulative Incidence of ADRD Diagnosis and Diagnostic Assessments at the Clinic Level eTable 2. Generalized Linear Mixed Model Results: Intervention Effect (by Screening Results) on ADRD Diagnosis and Diagnostic Assessments During a 12-Month Follow-Up eTable 3. Mixed-Effects Cox Model Results: Intervention Effect (by Screening Results) on Time to ADRD Diagnosis and Diagnostic Assessments During a 12-Month Follow-Up [file jamanetwopen-e2542222-s002.pdf]

Supplementary Online Content

Boustani MA, Ben Miled Z, Owora AH, et al. Digital detection of dementia in primary care: a randomized clinical trial. *JAMA Netw Open.* 2025;8(11):e2542222. doi:10.1001/jamanetworkopen.2025.42222

eFigure 1. Cumulative Incidence of Completed ADRD Diagnostic Assessments by Study Arm During a 12-Month Follow-Up
eFigure 2. Cumulative Incidence of Completed ADRD Diagnostic Assessments by Study Arm During a 12-Month Follow-Up Period by Type of Diagnostic Assessment
eAppendix. The Computerized Decision Support Workflow and Display
eFigure 3. The In-Basket Message to the Primary Care Clinician
eTable 1. Intervention Effect on the Cumulative Incidence of ADRD Diagnosis and Diagnostic Assessments at the Clinic Level
eTable 2. Generalized Linear Mixed Model Results: Intervention Effect (by Screening Results) on ADRD Diagnosis and Diagnostic Assessments During a 12-Month Follow-Up
eTable 3. Mixed-Effects Cox Model Results: Intervention Effect (by Screening Results) on Time to ADRD Diagnosis and Diagnostic Assessments During a 12-Month Follow-Up

This supplementary material has been provided by the authors to give readers additional information about their work.

© 2025 Boustani MA et al. *JAMA Network Open*.

eFigure 1. Cumulative Incidence of Completed ADRD Diagnostic Assessments by Study Arm During a 12-Month Follow-Up

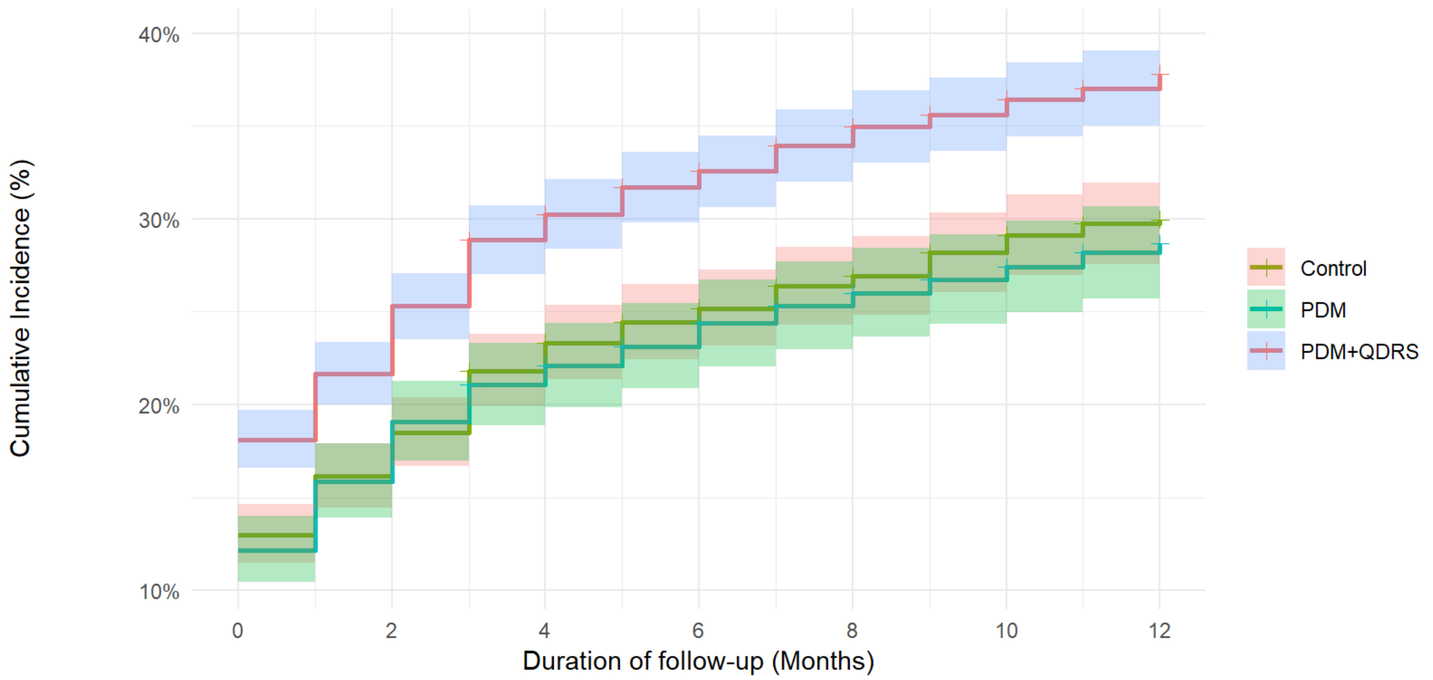

	0	2	4	6	8	10	12
**At Risk**							
Control	1724	1446	1336	1185	1078	990	911
PDM	1300	1094	1016	928	848	778	702
PDM+QDRS	2301	1803	1626	1434	1288	1158	1035
**Events**							
Control	224	319	402	432	458	489	500
PDM	158	248	287	315	334	349	362
PDM+QDRS	417	582	696	746	794	821	844

Gray's test: p<.0001

© 2025 Boustani MA et al. *JAMA Network Open*.

eFigure 2. Cumulative Incidence of Completed ADRD Diagnostic Assessments by Study Arm During a 12-Month Follow-Up Period by Type of Diagnostic Assessment

Laboratory Tests

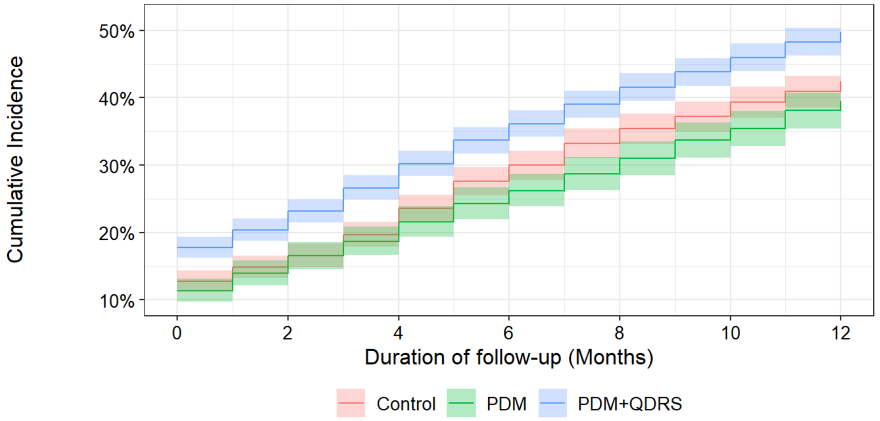

At Risk

	0	2	4	6	8	10	12
Control	1724	1468	1385	1246	1148	1073	1002
PDM	1300	1119	1056	981	915	847	779
PDM+QDRS	2301	1831	1688	1520	1391	1274	1155

Events

	0	2	4	6	8	10	12
Control	220	285	406	517	610	678	731
PDM	148	215	281	341	402	459	511
PDM+QDRS	409	533	696	832	956	1056	1141

Gray’s test: p<0.001

Imaging Tests

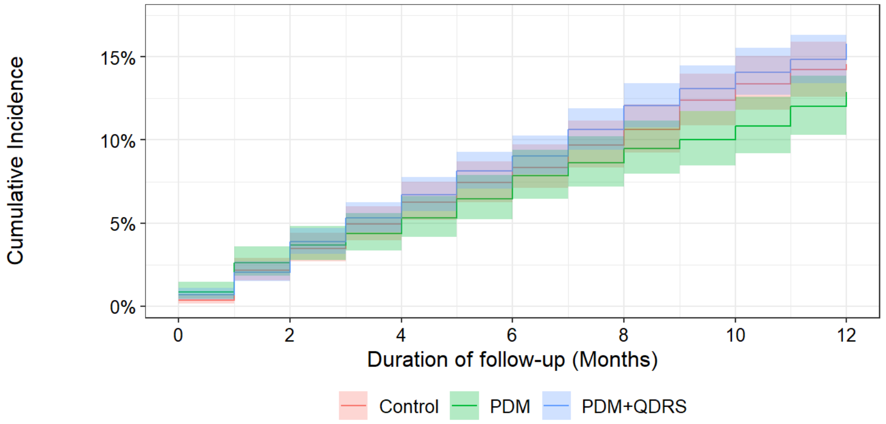

At Risk

	0	2	4	6	8	10	12
Control	1724	1687	1639	1591	1544	1488	1443
PDM	1300	1266	1242	1212	1169	1145	1106
PDM+QDRS	2301	2254	2179	2107	2038	1971	1895

Events

	0	2	4	6	8	10	12
Control	6	60	108	144	183	230	250
PDM	11	48	69	102	123	140	166
PDM+QDRS	16	89	154	208	277	323	361

Gray’s test: p=0.062

Only 1 PDM patient completed neuropsychological/neuropsychiatric testing within 12-months of enrollment.

Only 1 PDM+QDRS patient received ADRD medications within 12-months of enrollment.

© 2025 Boustani MA et al. *JAMA Network Open*.

eAppendix. The Computerized Decision Support Workflow and Display

Where will your patient access the Quick Rating Dementia Scale (QDRS)?

E-Check-in Walk Through

In MyChart, they will see their upcoming appt and select eCheck-In.

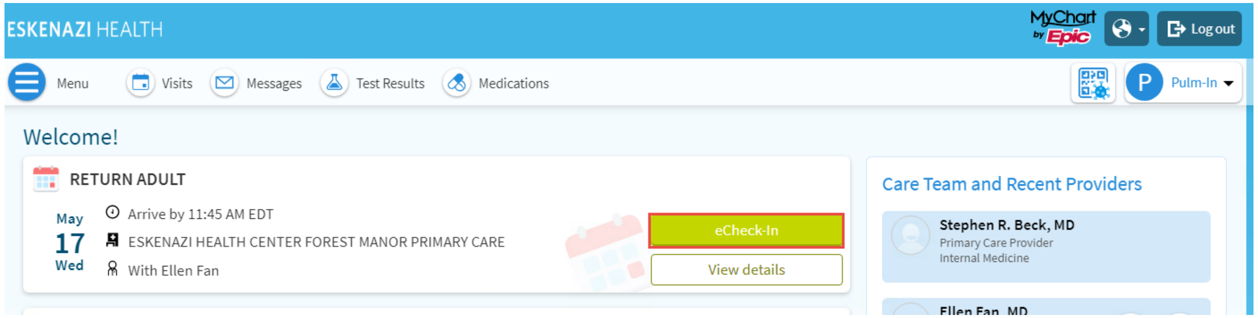

They will verify/update Personal Info, Contacts, Travel History, and Then carryout their **Questionnaires**

© 2025 Boustani MA et al. *JAMA Network Open*.

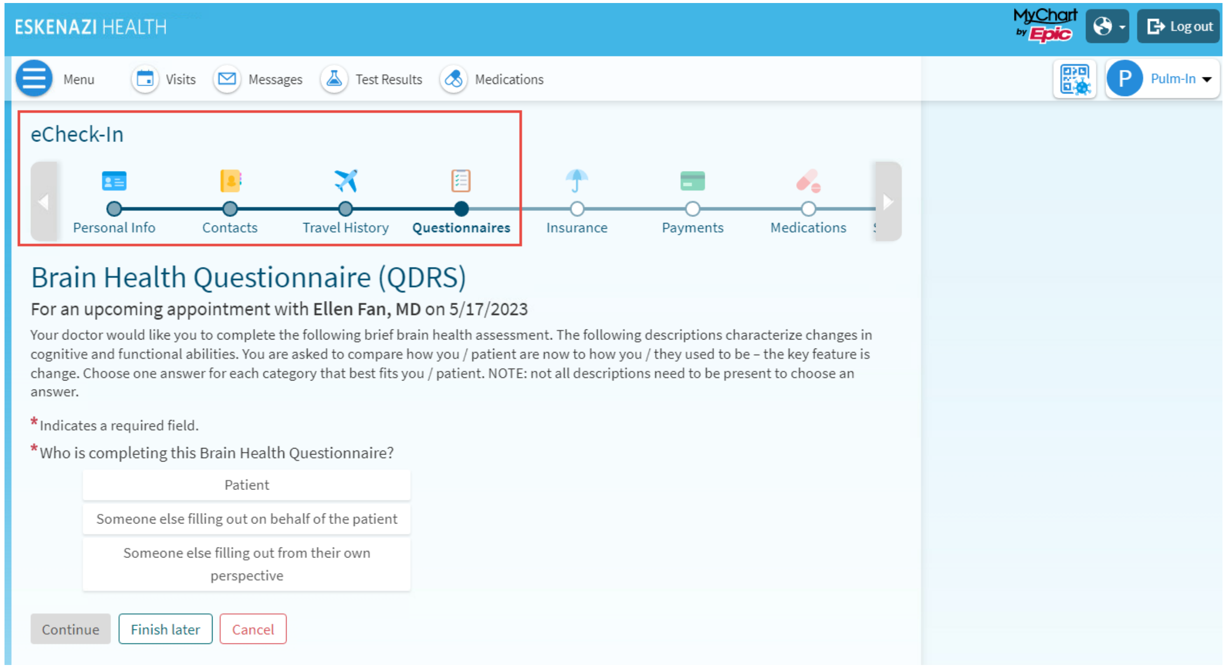

With each selection the Questionnaire will cascade.

© 2025 Boustani MA et al. *JAMA Network Open*.

*1. MEMORY AND RECALL

- No obvious memory loss or inconsistent forgetfulness that does not interfere with function in everyday activities
- Consistent mild forgetfulness or partial recollection of events that may interfere with performing everyday activities, repeats questions/statements, misplaces items, forgets appointments
- Mild to moderate memory loss, more noticeable for recent events, interferes with performing everyday activities
- Moderate to severe memory loss, only highly learned information remembered, new information rapidly forgotten
- Severe memory loss, almost impossible to recall new information, long-term memory may be affected

*2. ORIENTATION

- Fully oriented to person, place, and time nearly all the time
- Slight difficulty keeping track of time, may forget day or date more frequently than in the past
- Mild to moderate Mild to moderate difficulty keeping track of time and sequence of events, forgets month or year, oriented to familiar places but gets confused outside of familiar areas, gets lost or wanders
- Moderate to severe difficulty, usually disoriented to time and place (familiar and unfamiliar), frequently dwells in past
- Only oriented to their name, although may recognize family members

*3. DECISION MAKING AND PROBLEM SOLVING ABILITIES

- Fully oriented to person, place, and time nearly all the time
- Slight difficulty keeping track of time, may forget day or date more frequently than in the past
- Mild to moderate Mild to moderate difficulty keeping track of time and sequence of events, forgets month or year, oriented to familiar places but gets confused outside of familiar areas, gets lost or wanders
- Moderate to severe difficulty, usually disoriented to time and place (familiar and unfamiliar), frequently dwells in past
- Only oriented to their name, although may recognize family members

*4. ACTIVITIES OUTSIDE THE HOME

- Independent in function at usual level of performance in profession, shopping, community and religious activities, volunteering, or social groups
- Slight impairment in these activities compared to previous performance, slight change in driving skills, still able to handle emergency situations
- Unable to function independently but still may attend and be engaged, appears "normal" to others, notable changes in driving skills, concern about ability to handle emergency situations
- No pretense of independent function outside the home, appears well enough to be taken to activities outside the family home but generally needs to be accompanied
- No independent function or activities, appear too ill to be taken to activities outside the home

*5. FUNCTION AT HOME AND HOBBY ACTIVITIES

- Chores at home, hobbies and personal interests are well maintained compared to past performance
- Slight impairment or less interest in these activities, trouble operating appliances (particularly new purchases)
- Mild but definite impairment in home and hobby function, more difficult chores or tasks abandoned, more complicated hobbies and interests given up
- Only simple chores preserved, very restricted interest in hobbies which are poorly maintained
- No meaningful function in household chores or with prior hobbies

*6. TOILETING AND PERSONAL HYGEINE

- Fully capable of self-care (dressing, grooming, washing, bathing, toileting)
- Slight changes in abilities and attention to these activities
- Needs prompting to complete these activities but may still complete independently
- Requires some assistance in dressing, hygiene, keeping of personal items, occasionally incontinent
- Requires significant help with personal care and hygiene: frequent incontinence

© 2025 Boustani MA et al. *JAMA Network Open*.

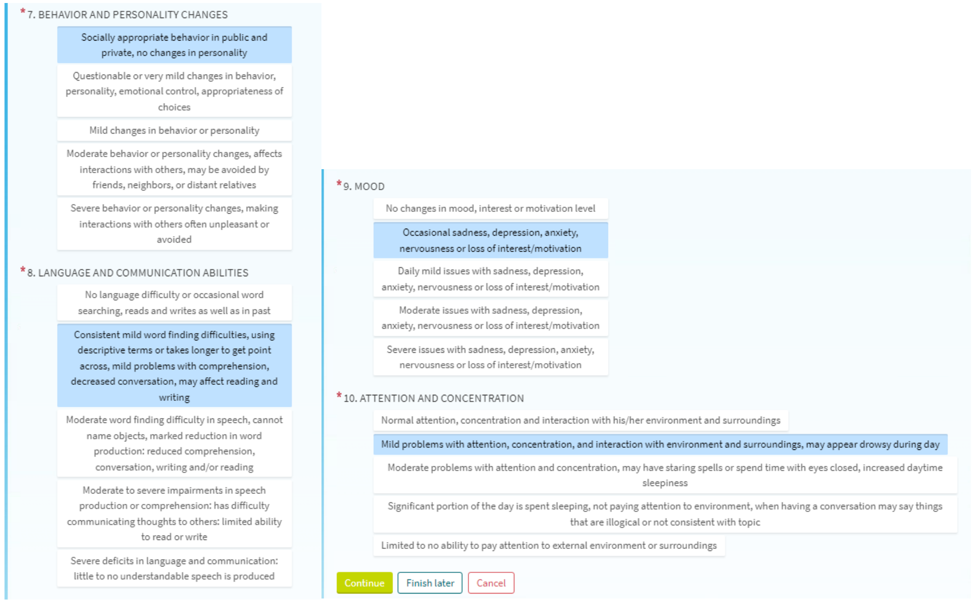

The patient will be able to review and go back and edit their answers if needed. Then they will submit the questionnaire.

© 2025 Boustani MA et al. *JAMA Network Open*.

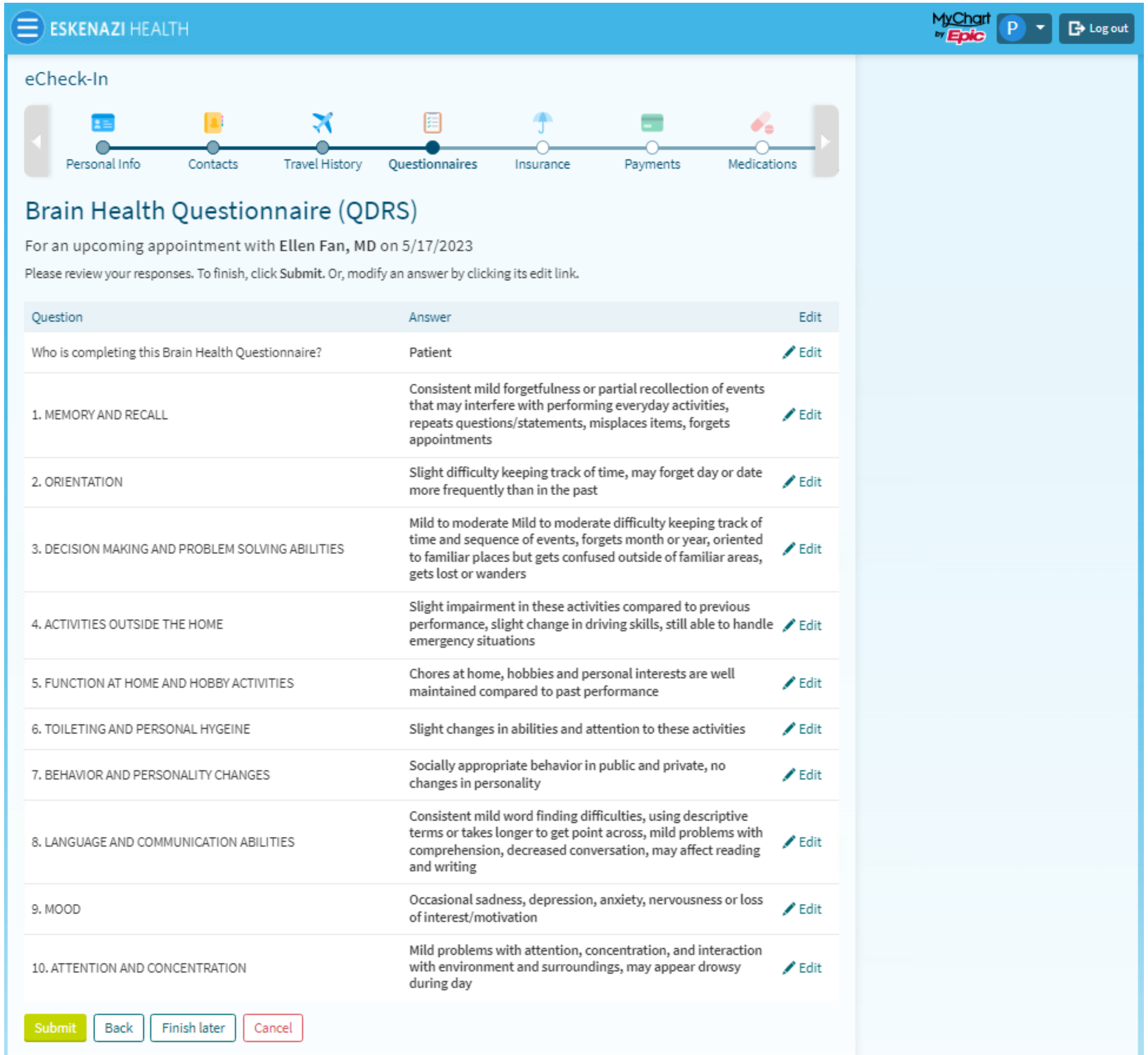

ESKENAZI HEALTH

MyChart by Epic

P

Log out

eCheck-In

Personal Info | Contacts | Travel History | Questionnaires | Insurance | Payments | Medications

Brain Health Questionnaire (QDRS)

For an upcoming appointment with **Ellen Fan, MD** on 5/17/2023

Please review your responses. To finish, click **Submit**. Or, modify an answer by clicking its edit link.

Question	Answer	Edit
Who is completing this Brain Health Questionnaire?	Patient	Edit
1. MEMORY AND RECALL	Consistent mild forgetfulness or partial recollection of events that may interfere with performing everyday activities, repeats questions/statements, misplaces items, forgets appointments	Edit
2. ORIENTATION	Slight difficulty keeping track of time, may forget day or date more frequently than in the past	Edit
3. DECISION MAKING AND PROBLEM SOLVING ABILITIES	Mild to moderate Mild to moderate difficulty keeping track of time and sequence of events, forgets month or year, oriented to familiar places but gets confused outside of familiar areas, gets lost or wanders	Edit
4. ACTIVITIES OUTSIDE THE HOME	Slight impairment in these activities compared to previous performance, slight change in driving skills, still able to handle emergency situations	Edit
5. FUNCTION AT HOME AND HOBBY ACTIVITIES	Chores at home, hobbies and personal interests are well maintained compared to past performance	Edit
6. TOILETING AND PERSONAL HYGEINE	Slight changes in abilities and attention to these activities	Edit
7. BEHAVIOR AND PERSONALITY CHANGES	Socially appropriate behavior in public and private, no changes in personality	Edit
8. LANGUAGE AND COMMUNICATION ABILITIES	Consistent mild word finding difficulties, using descriptive terms or takes longer to get point across, mild problems with comprehension, decreased conversation, may affect reading and writing	Edit
9. MOOD	Occasional sadness, depression, anxiety, nervousness or loss of interest/motivation	Edit
10. ATTENTION AND CONCENTRATION	Mild problems with attention, concentration, and interaction with environment and surroundings, may appear drowsy during day	Edit

Submit | Back | Finish later | Cancel

© 2025 Boustani MA et al. *JAMA Network Open*.

Once submitted, the **Provider** can see the patient's questionnaire/answers/Score in the Rooming Activity>Questionnaire Section. BPA will prompt if eligible score is met.

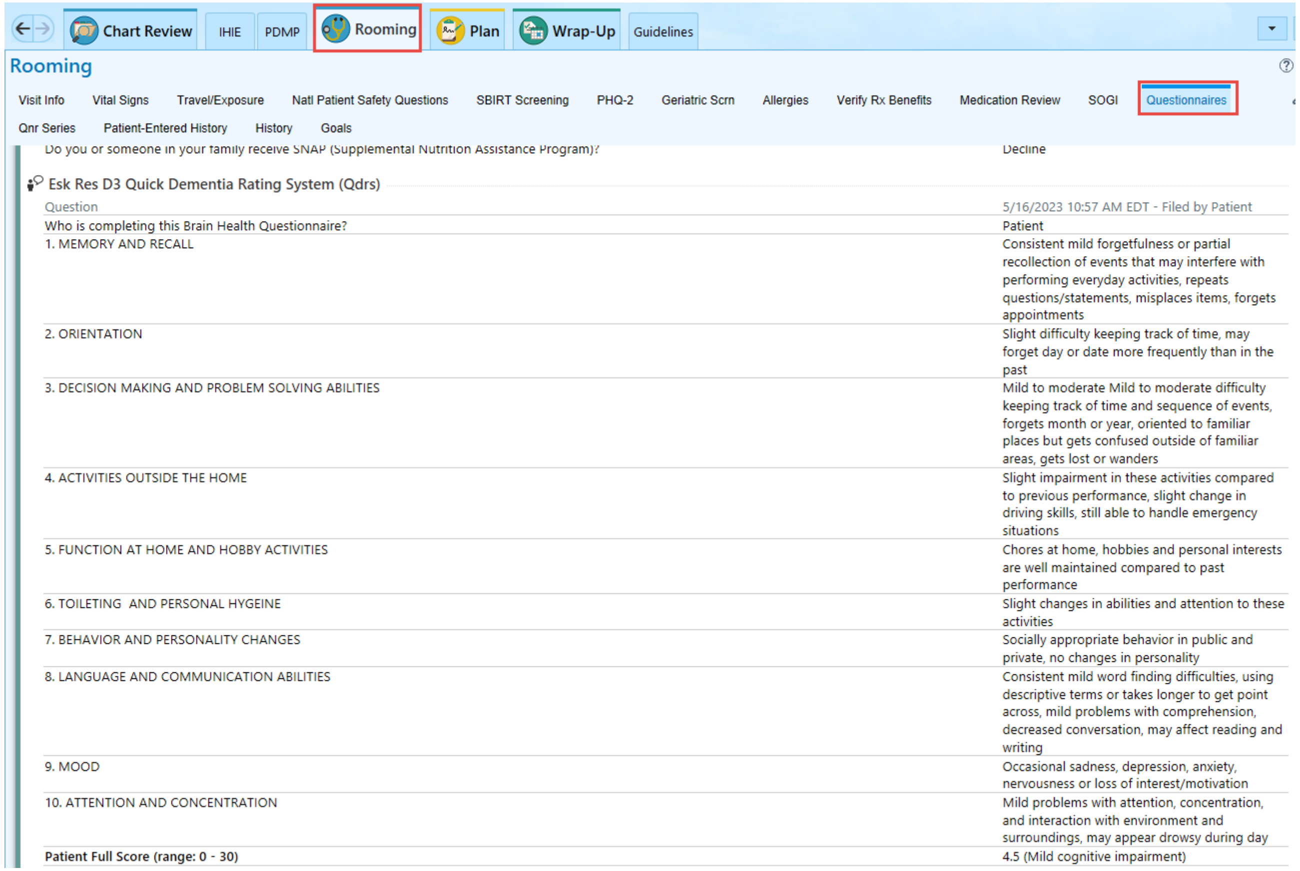
Chart Review | IHIE | PDMP | Rooming | Plan | Wrap-Up | Guidelines

Rooming

Visit Info | Vital Signs | Travel/Exposure | Natl Patient Safety Questions | SBIRT Screening | PHQ-2 | Geriatric Scrn | Allergies | Verify Rx Benefits | Medication Review | SOGI | Questionnaires

Qnr Series | Patient-Entered History | History | Goals

Do you or someone in your family receive SNAP (Supplemental Nutrition Assistance Program)? — Decline

Esk Res D3 Quick Dementia Rating System (Qdrs)

Question	5/16/2023 10:57 AM EDT - Filed by Patient
Who is completing this Brain Health Questionnaire?	Patient
1. MEMORY AND RECALL	Consistent mild forgetfulness or partial recollection of events that may interfere with performing everyday activities, repeats questions/statements, misplaces items, forgets appointments
2. ORIENTATION	Slight difficulty keeping track of time, may forget day or date more frequently than in the past
3. DECISION MAKING AND PROBLEM SOLVING ABILITIES	Mild to moderate Mild to moderate difficulty keeping track of time and sequence of events, forgets month or year, oriented to familiar places but gets confused outside of familiar areas, gets lost or wanders
4. ACTIVITIES OUTSIDE THE HOME	Slight impairment in these activities compared to previous performance, slight change in driving skills, still able to handle emergency situations
5. FUNCTION AT HOME AND HOBBY ACTIVITIES	Chores at home, hobbies and personal interests are well maintained compared to past performance
6. TOILETING AND PERSONAL HYGEINE	Slight changes in abilities and attention to these activities
7. BEHAVIOR AND PERSONALITY CHANGES	Socially appropriate behavior in public and private, no changes in personality
8. LANGUAGE AND COMMUNICATION ABILITIES	Consistent mild word finding difficulties, using descriptive terms or takes longer to get point across, mild problems with comprehension, decreased conversation, may affect reading and writing
9. MOOD	Occasional sadness, depression, anxiety, nervousness or loss of interest/motivation
10. ATTENTION AND CONCENTRATION	Mild problems with attention, concentration, and interaction with environment and surroundings, may appear drowsy during day
**Patient Full Score (range: 0 - 30)**	4.5 (Mild cognitive impairment)

© 2025 Boustani MA et al. *JAMA Network Open*.

<u>Where will you find the PDM notification if your patient has an Abnormal Dementia Screen?</u>

Storyboard

The PDM alert will be in your **Storyboard** when you open the patient’s chart review, highlighted in yellow.

© 2025 Boustani MA et al. *JAMA Network Open*.

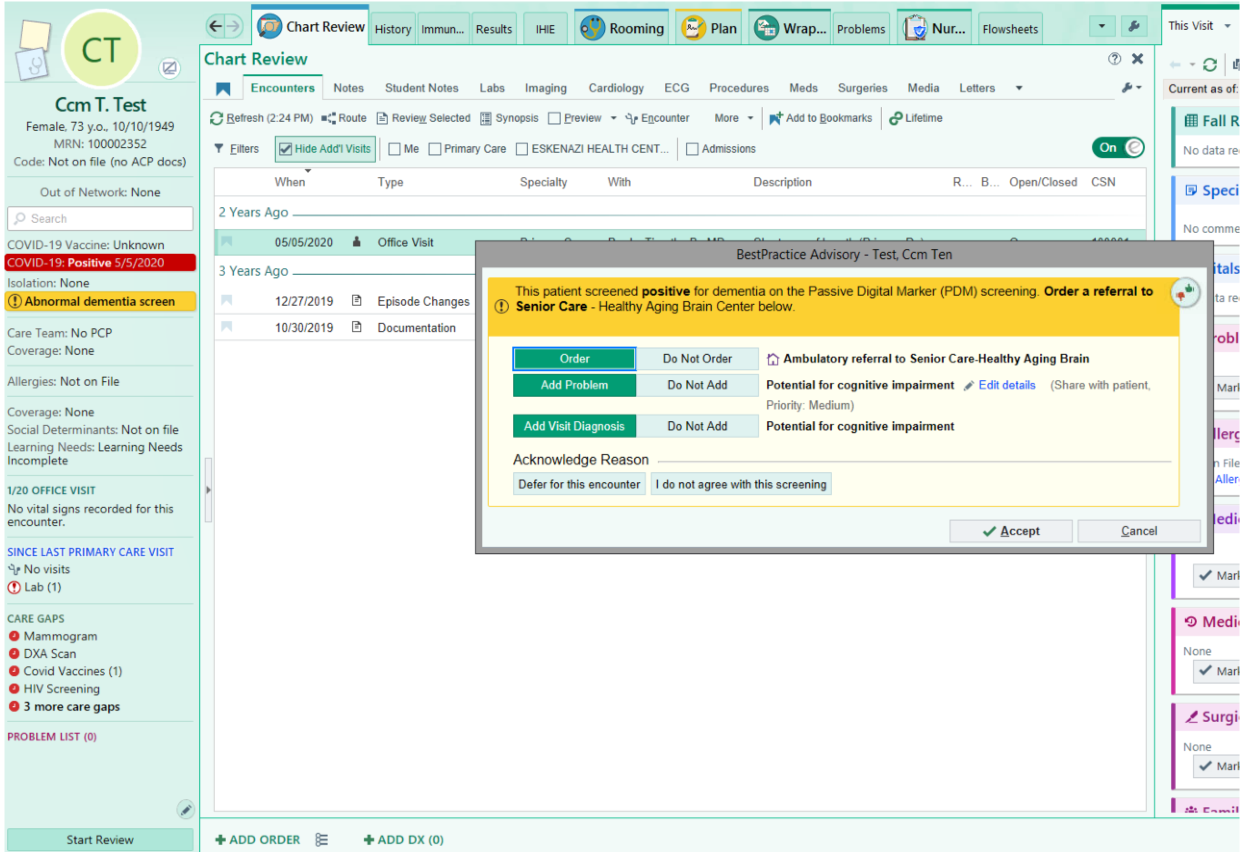

When you click on the notification, the **Non-Interruptive Alert** will open and give you four options. The first three options give you the opportunity to easily add or to not add a referral to the Healthy Aging Brain Center and/or to add or not add documentation for a potential for cognitive impairment:

1. Order/Do not Order an **Ambulatory referral to Senior Care-Healthy Aging Brain**
2. Add Problem/Do Not Add **Potential for cognitive impairment**
3. Add Visit Diagnosis/Do Not Add **Potential for cognitive impairment**

© 2025 Boustani MA et al. *JAMA Network Open*.

The fourth choice gives you two options:

4. Defer the BPA until your patient's next office visit
5. Document that you disagree with the Abnormal Dementia Screen decision made by the PDM

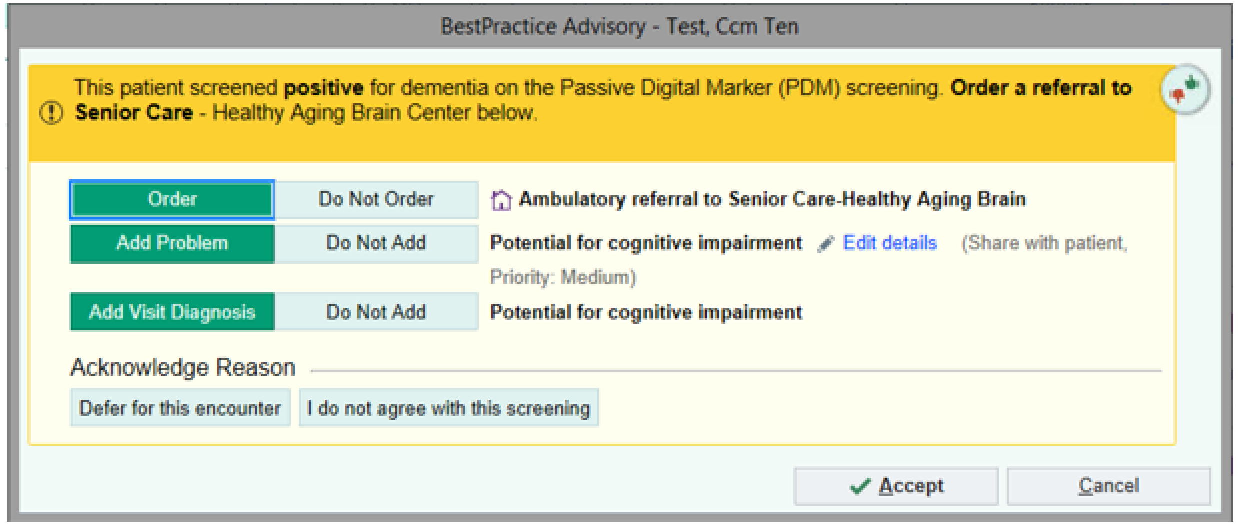

© 2025 Boustani MA et al. *JAMA Network Open*.

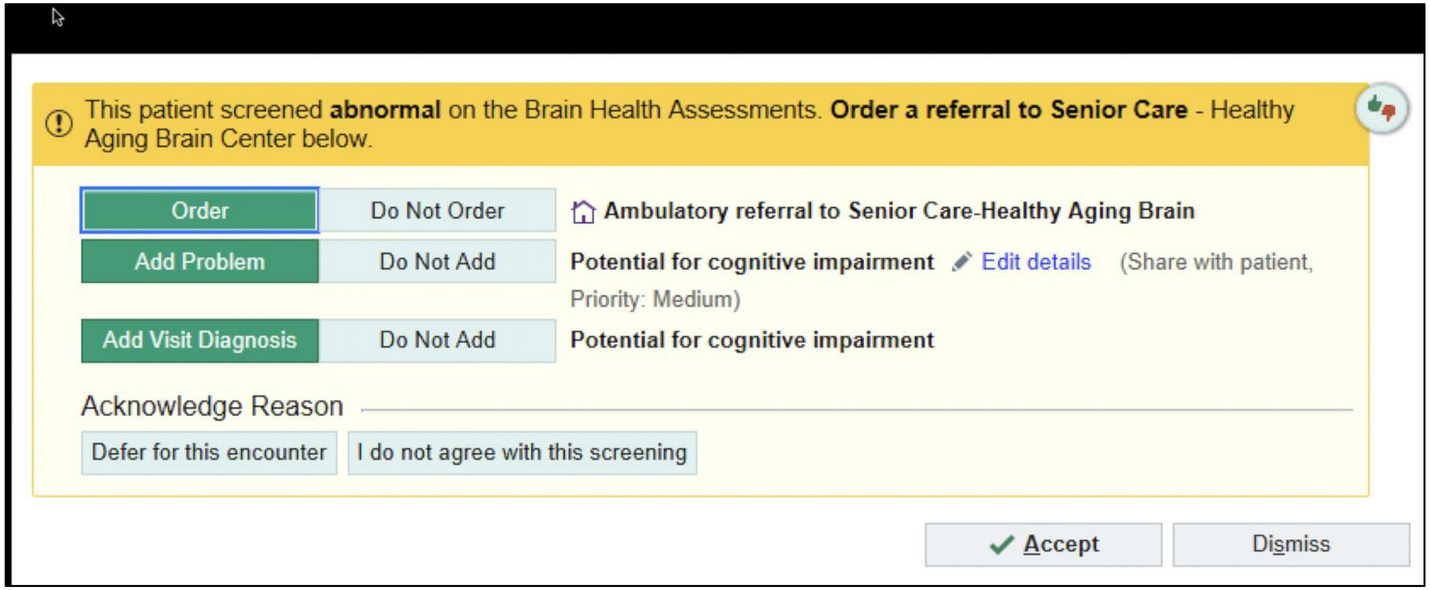

© 2025 Boustani MA et al. *JAMA Network Open*.

eFigure 3. The In-Basket Message to the Primary Care Clinician

Dear [Dr. PCP],<br>*Thank you for supporting brain care at Eskenazi Health.*<br>*An algorithm embedded in Epic has detected that your patient, ___________, is at risk for cognitive impairment due to the following factors:*<br>*1.*<br>*2.*<br>*3.*<br>*They have also completed a patient reported questionnaire on cognitive symptoms by the QDRS. Your patient's answer(s) to the following question(s) may mean cognitive impairment.:*<br>*1.*<br>*2.*<br>*3.*<br><br>*Please consider referring your patient to the Healthy Aging Brain Center if it seems clinically appropriate.*<br>*If you feel that your patient was detected in error, please respond to this message and let us know.*<br>*Thank you for your time and contribution,*

© 2025 Boustani MA et al. *JAMA Network Open*.

eTable 1. Intervention Effect on the Cumulative Incidence of ADRD Diagnosis and Diagnostic Assessments at the Clinic Level

	**Control**	**PDM Effect**			**PDM+QDRS Effect**		
Patient sample size (%)	1,724 (32.4%)	1,300 (24.4%)			2,301 (43.2%)		
Number of clinics	3	3			3		
	Mean Cumulative Incidence (95% CI)	Mean Cumulative Incidence (95%CI)	mean difference (95% CI)	P value	Mean Cumulative Incidence (95% CI)	mean difference (95%CI)	P value
**Primary Outcome**							
ADRD Diagnosis	12.4 (10.3, 14.4)	10.3 (7.96, 12.7)	-2.05 (-5.15, 1.06)	0.158	15.4 (13.7, 17.2)	3.07 (0.38, 5.77)	0.032
**Secondary Outcome**							
ADRD Diagnostic Assessments	29.0 (24.5, 33.5)	27.8 (22.7, 33.0)	-1.16 (-8.03, 5.72)	0.695	36.7 (32.8, 40.6)	7.68 (1.72, 13.6)	0.020

Mean cumulative incidence estimates are derived from a weighted linear regression model (cluster size used as weights) and mean differences are based on post hoc comparisons with Tukey HSD adjustment for multiple group comparisons.

ADRD diagnosis model: yielded an R-squared of 0.77, indicating that 77% of the variability in ADRD diagnoses is explained by study arm. The model was statistically significant (F (2,6) = 9.853, p =0.0127).

ADRD diagnostic services model: yielded an R-squared of 0.71, indicating that 71% of the variability in ADRD diagnostic assessments is explained by study arm. The model was statistically significant (F (2,6) = 7.555, p =0.0229).

© 2025 Boustani MA et al. *JAMA Network Open*.

eTable 2. Generalized Linear Mixed Model Results: Intervention Effect (by Screening Results) on ADRD Diagnosis and Diagnostic Assessments During a 12-Month Follow-Up

	Study arm									
	Control		PDM		PDM+QDRS					
Screening result	PDM-	PDM+	PDM-	PDM+	PDM-QDRS-	PDM-QDRS+	PDM-QDRS missing	PDM+QDRS-	PDM+QDRS+	PDM+QDRS missing
Patient sample size (%)	1,163 (21.8%)	561 (10.5%)	902 (16.9%)	398 (7.5%)	282 (5.3%)	41 (0.8%)	1,162 (21.8%)	115 (2.2%)	38 (0.7%)	663 (12.5%)
**Diagnosis: cumulative incidence** (%)	126 (10.8%)	87 (15.5%)	64 (7.1%)	70 (17.6%)	25 (8.9%)	13 (31.7%)	131 (11.3%)	15 (13.0%)	12 (31.6%)	159 (24.0%)
Crude odds ratio (95% CI)	Reference	1.51 (1.06, 2.15)	0.63 (0.43, 0.92)	1.76 (1.20, 2.58)	0.80 (0.47, 1.38)	3.82 (1.68, 8.71)	1.05 (0.76, 1.43)	1.23 (0.62, 2.47)	3.80 (1.62, 8.93)	2.60 (1.90, 3.54)
P value	Reference	0.006	0.004	<0.001	0.333	<0.001	0.735	0.471	<0.001	<0.001
Adjusted odds ratio (95% CI)	Reference	1.41 (0.98, 2.01)	0.65 (0.44, 0.95)	1.63 (1.11, 2.41)	0.81 (0.47, 1.40)	3.85 (1.68, 8.82)	1.06 (0.77, 1.45)	1.19 (0.59, 2.39)	3.47 (1.47, 8.23)	2.44 (1.78, 3.33)
P value	Reference	0.024	0.007	0.003	0.371	<0.001	0.679	0.550	<0.001	<0.001
**Diagnostic Assessments: cumulative Incidence** (%)	304 (26.1%)	196 (34.9%)	218 (24.2%)	144 (36.2%)	82 (29.1%)	20 (48.8%)	400 (34.4%)	48 (41.7%)	19 (50.0%)	275 (41.5%)
Crude odds ratio (95% CI)	Reference	1.50 (1.15, 1.95)	0.90 (0.67, 1.21)	1.61 (1.15, 2.25)	1.14 (0.78, 1.67)	2.67 (1.24, 1.92)	1.48 (1.13, 1.92)	1.98 (1.20, 3.27)	2.77 (1.25, 6.17)	1.98 (1.49, 2.65)
P value	Reference	<0.001	0.414	<0.001	0.429	0.003	<0.001	0.001	0.003	<0.001
Adjusted odds ratio (95% CI)	Reference	1.48 (1.14, 1.93)	0.89 (0.67, 1.19)	1.54 (1.11, 2.14)	1.12 (0.77, 1.63)	2.65 (1.23, 5.73)	1.47 (1.14, 1.90)	1.94 (1.18, 3.18)	2.58 (1.16, 5.73)	1.96 (1.48, 2.49)
P value	Reference	<0.001	0.349	0.002	0.493	0.003	<0.001	0.002	0.005	<0.001

Generalized Linear Mixed-Effects models include a between-within correction to maintain appropriate Type I error and Tukey multiple comparison adjustments to control the

© 2025 Boustani MA et al. *JAMA Network Open*.

family-wise error rate (FWER).

Adjusted odds ratio includes covariate adjustment for differences in age, sex, and race distribution.

Furthermore, our Per-protocol analyses (NOT intent to treat) based on study arm and screening results (from the PDM and QDRS) showed that irrespective of study arm, patients who had a PDM positive screening result had a higher cumulative incidence (odds) of ADRD diagnostic assessments than patients in the control arm with a negative PDM screening result. Having a positive QDRS result (irrespective of PDM results ±) was also associated with higher odds of ADRD diagnostic assessments. Both results help explain the higher cumulative incidence of ADRD diagnoses in the combined intervention arms.

eTable 3. Mixed-Effects Cox Model Results: Intervention Effect (by Screening Results) on Time to ADRD Diagnosis and Diagnostic Assessments During a 12-Month Follow-Up

	Study arm									
	Control		PDM		PDM+QDRS					
Screening Results	PDM-	PDM+	PDM-	PDM+	PDM-QDRS-	PDM-QDRS+	PDM-QDRS missing	PDM+QDRS-	PDM+QDRS+	PDM+QDRS missing
Patient sample size (%)	1,163 (21.8%)	561 (10.5%)	902 (16.9%)	398 (7.5%)	282 (5.3%)	41 (0.8%)	1,162 (21.8%)	115 (2.2%)	38 (0.7%)	663 (12.5%)
**Diagnosis: cumulative incidence** (%)	126 (10.8%)	87 (15.5%)	64 (7.1%)	70 (17.6%)	25 (8.9%)	13 (31.7%)	131 (11.3%)	15 (13.0%)	12 (31.6%)	159 (24.0%)
Crude hazard ratio (95% CI)	Reference	1.59 (1.15, 2.20)	1.00 (0.69, 1.45)	1.95 (1.38, 2.78)	1.03 (0.62, 1.71)	4.95 (2.65, 9.23)	1.21 (0.89, 1.63)	1.44 (0.76, 2.72)	2.33 (1.20, 4.51)	2.71 (2.04, 3.58)
P value	Reference	0.005	0.990	<0.001	0.909	<0.001	0.220	0.265	0.012	<0.001
Adjusted hazard ratio (95% CI)	Reference	1.38 (1.00, 1.90)	1.05 (0.73, 1.52)	1.75 (1.23, 2.48)	1.05 (0.63, 1.75)	4.44 (2.42, 8.14)	1.13 (0.84, 1.53)	1.33 (0.71, 2.49)	1.86 (0.94, 3.68)	2.28 (1.72, 3.01)
P value	Reference	0.052	0.780	0.002	0.846	<0.001	0.410	0.380	0.075	<0.001
**Diagnostic services: cumulative Incidence** (%)	304 (26.1%)	196 (34.9%)	218 (24.2%)	144 (36.2%)	82 (29.1%)	20 (48.8%)	400 (34.4%)	48 (41.7%)	19 (50.0%)	275 (41.5%)
Crude hazard ratio (95% CI)	Reference	1.53 (1.23, 1.91)	0.93 (0.76, 1.14)	1.50 (1.20, 1.88)	1.09 (0.83, 1.43)	2.52 (1.56, 4.06)	1.39 (1.17, 1.65)	1.86 (1.34, 2.58)	2.15 (1.41, 3.27)	1.79 (1.48, 2.17)
P value	Reference	<0.001	0.474	<0.001	0.538	<0.001	<0.001	<0.001	<0.001	<0.001
Adjusted hazard ratio (95% CI)	Reference	1.50 (1.21, 1.86)	0.92 (0.75, 1.13)	1.42 (1.13, 1.79)	1.08 (0.83, 1.42)	2.50 (1.54, 4.06)	1.38 (1.16, 1.64)	1.84 (1.33, 2.55)	2.01 (1.33,	1.73 (1.43, 2.10)

© 2025 Boustani MA et al. *JAMA Network Open*.

									3.04)	
P value	Reference	<0.001	0.445	0.002	0.562	<0.001	<0.001	<0.001	<0.001	<0.001

Mixed-Effects Cox models include Tukey multiple comparison adjustments to control the family-wise error rate (FWER).

Adjusted odds ratio includes covariate adjustment for differences in age, sex, and race distribution.

© 2025 Boustani MA et al. *JAMA Network Open*.
