# Supplementary material for: Digital Detection of Dementia in Primary Care: A Randomized Clinical Trial
Source: JAMA Netw Open. 2025 Nov 10;8(11):e2542222. doi: 10.1001/jamanetworkopen.2025.42222 (PMC12603861; doi:10.1001/jamanetworkopen.2025.42222)
Supplement: Supplement 3. — Data Sharing Statement [file jamanetwopen-e2542222-s003.pdf]

Data Sharing Statement

Boustani. Digital Detection of Dementia in Primary Care. *JAMA Netw Open*. Published November 10, 2025. doi:10.1001/jamanetworkopen.2025.42222

Data
Additional Information: Digital Detection of Dementia (D cubed) Studies: D2. NCT05231954 https://register.clinicaltrials.gov/prs/beta/studies/S000BJ5B00000092/recordSummary
Data available: No
